# TP63–TRIM29 axis regulates enhancer methylation and chromosomal instability in prostate cancer

**DOI:** 10.1186/s13072-024-00529-7

**Published:** 2024-03-14

**Authors:** R. Sultanov, A. Mulyukina, O. Zubkova, A. Fedoseeva, A. Bogomazova, K. Klimina, A. Larin, T. Zatsepin, T. Prikazchikova, M. Lukina, M. Bogomiakova, E. Sharova, E. Generozov, M. Lagarkova, G. Arapidi

**Affiliations:** 1 Center for Precision Genome Editing and Genetic Technologies for Biomedicine, Lopukhin Federal Research and Clinical Center of Physical-Chemical Medicine of Federal Medical Biological Agency, Moscow, Russia; 2 Lopukhin Federal Research and Clinical Center of Physical-Chemical Medicine of Federal Medical Biological Agency, Moscow, Russia; 3 Shemyakin-Ovchinnikov Institute of Bioorganic Chemistry of the Russian Academy of Sciences, Moscow, Russia; 4 Department of Chemistry, Lomonosov Moscow State University, Moscow, Russia

**Keywords:** TP63, TRIM29, Epigenetic regulation, Prostate cancer, WGCNA

## Abstract

**Background:**

Prostate adenocarcinoma (PRAD) is the second leading cause of cancer-related deaths in men. High variability in DNA methylation and a high rate of large genomic rearrangements are often observed in PRAD.

**Results:**

To investigate the reasons for such high variance, we integrated DNA methylation, RNA-seq, and copy number alterations datasets from The Cancer Genome Atlas (TCGA), focusing on PRAD, and employed weighted gene co-expression network analysis (WGCNA). Our results show that only single cluster of co-expressed genes is associated with genomic and epigenomic instability. Within this cluster, TP63 and TRIM29 are key transcription regulators and are downregulated in PRAD. We discovered that TP63 regulates the level of enhancer methylation in prostate basal epithelial cells. TRIM29 forms a complex with TP63 and together regulates the expression of genes specific to the prostate basal epithelium. In addition, TRIM29 binds DNA repair proteins and prevents the formation of the *TMPRSS2:ERG* gene fusion typically observed in PRAD.

**Conclusion:**

Our study demonstrates that TRIM29 and TP63 are important regulators in maintaining the identity of the basal epithelium under physiological conditions. Furthermore, we uncover the role of TRIM29 in PRAD development.

**Supplementary Information:**

The online version contains supplementary material available at 10.1186/s13072-024-00529-7.

## Background

Prostate adenocarcinoma (PRAD) is the most frequently diagnosed cancer in men and the second leading cause of cancer mortality affecting men worldwide [1]. In contrast to other types of cancer, PRAD is characterized by a low number of recurrent single nucleotide variations (SNVs). At the same time, it exhibits high genomic and chromosomal instability accompanied by a high frequency of gene fusions (e.g., *TMPRSS2:ERG* is found in ~ 50% of patients) and high epigenomic variability [2–4]. Many studies have demonstrated that epigenetic changes, including DNA methylation and histone modifications, are required for the initiation and progression of PRAD [5–7]. Methylome changes are often caused by somatic mutations in genes of the DNA methylation-demethylation system and chromatin modulator system (*DNMT3B*, *TET2*, *BRAF*, *IDH1*) [8]. Altering the activity of transcription factors such as androgen receptor (AR) or ETS-related gene (ERG) can also lead to dramatic changes in the epigenetic landscape [9]. Interestingly, changes in methylation patterns may also affect the activity of genes involved in cell cycle control, response to hormones, and DNA damage repair [8, 10, 11]. Chromosomal instability in PRAD is often the result of a faulty DNA damage repair system, leading to amplification and deletion of large loci and chromosome arms [3, 4, 12]. PRAD exhibits amplification of such oncogenes as MYC and AR and the formation of the *TMPRSS2:ERG* gene fusion, which is the most common recurrent somatic mutation in PRAD. *TMPRSS2:ERG* gene fusion has pioneering transcription factor properties that can alter the epigenetic landscape and cause (Babu and Fullwood. [13], Kron et al. [14]) MYC and AR overexpression [9, 15]. Thus, chromosomal instability and changes in DNA methylation can mutually influence each other. However, molecular mechanisms that lead to high chromosomal instability and epigenomic variability remain unclear. In recent years, continued efforts from several projects like The Cancer Genome Atlas (TCGA), International Cancer Genome Consortium (ICGC), Chinese Prostate Cancer Genome and Epigenome Atlas (CPGEA), and Taylor dataset [3] have helped gathering multiple PRAD-related omics data. Comprehensive profiling of RNA seq, DNA methylation, and somatic mutations data from these projects has revealed seven distinct subtypes of PRAD [4]. However, integrating various omics data and interpreting the results still present some challenges. RNA profiling is one of the significant genomic tools to study molecular mechanisms underlying various diseases. RNA-seq captures the average gene expression profile from the tissue. However, due to the highly heterogeneous nature of PRAD tumors, the resulting average gene expression profile from tumor tissues is not always a direct depiction of their signature molecular events. This issue can be addressed by searching for clusters (or modules) of co-expressed genes to be further associated with clinical data and phenotypes. Weighted gene co-expression network analysis (WGCNA) [16] allows assembly of thousands of genes with similar patterns of expression into clusters. These clusters can be associated with various phenotypes or other omics data for correlative or quantitative studies. The WGCNA approach has already been used to search for important gene hubs, biomarkers, therapeutic targets, or integration with omics data for PRAD [17–19]. In addition, the WGCNA algorithm has been proven to be an exemplary method for efficient generation of hypotheses [17–19].

Here, we used the WGCNA correlation network analysis algorithm to search for clusters of co-expressed genes associated with epigenetic variability and chromosomal instability in PRAD using the TCGA PRAD data set. Further analysis of the module using GO, TF-enrichment analysis, and KEGG analysis identified the TP63 cluster responsible for regulating the cluster of genes associated with epigenetic variability and genome instability in PRAD. We showed that TRIM29 interacts with TP63 and regulates the expression of the TP63 cluster, which is associated with variability in DNA methylation and chromosomal instability. Using *TP63* and *TRIM29* gene knockdown and overexpression in two cell lines of the normal basal epithelium of the prostate and two cell lines of PRAD, we showed that TP63 directly regulates methylation levels of CpG sites in enhancers specific for the basal epithelium of the prostate. In contrast, TRIM29 regulates the activity of transcription factor TP63. Furthermore, we show that in the TP63 cluster, TRIM29 performs the genome protection functions against chromosomal instability.

## Results

### TP63 regulates the gene cluster associated with epigenetic variability and chromosomal instability in PRAD

**Fig. 1:**
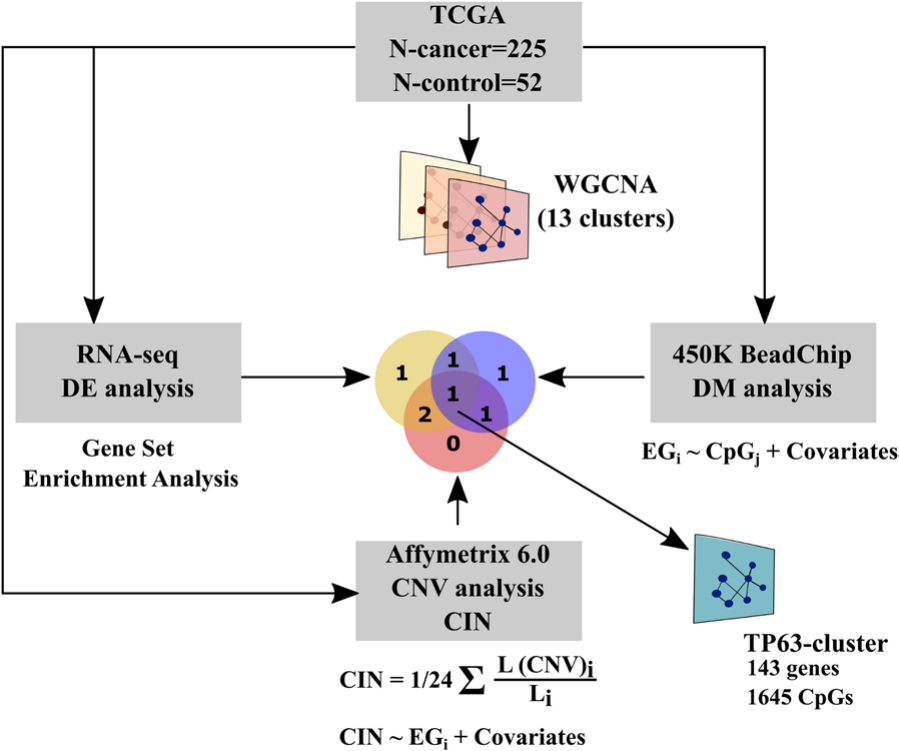
Clusters of co-expressed genes associated with epigenetic variability and chromosomal instability in PRAD. In brief, 13 clusters of co-expression genes were generated from TCGA PRAD datasets using WGCNA [16]. To identify clusters associated with epigenetic variability and chromosomal instability, GSEA, regression analysis between eigengene of cluster and CpG-site methylation or chromosomal instability index was performed. *DE* differential expression, *DM *differential methylation, *CIN* chromosomal instability index

To unveil a cluster of co-expressed genes associated with epigenomic variability and chromosomal instability, we analyzed RNA-seq, DNA methylation (Infinium MethylationBeadChip450K), and copy number variation (CNV, Affymetrix 6.0) datasets of The Cancer Genome Atlas (TCGA) for prostate adenocarcinoma (PRAD). In brief, we determined 13 co-expressed gene clusters in the RNA-seq data using the WGCNA package [16]. To determine association of clusters with differentially expressed genes between tumor and adjacent non-tumor tissues, we used Gene Set Enrichment Analysis (GSEA) [20]. Linear regression was applied to find association between eigengene of each cluster and methylation level of CpG-sites or chromosomal instability index (see Methods for details). As demonstrated in a previous work, the level of CpG site methylation can strongly depend on the ratio of various types of prostate cells in a sample [21]. To calculate the ratio of stromal, basal, and luminal epithelium cells in TCGA samples, we used prostate scRNA-seq data [22]. The calculated ratios of cell type composition were used as covariates for further regression analysis. We found only a single cluster of coexpressed genes (size of the cluster = 143 genes, Additional file 1: Table S2) that were simultaneously associated with the methylation level of CpG sites, chromosomal instability, and enriched with PRAD-associated genes (see Methods for details). This cluster exhibited strong correlations with the methylation levels of 1645 differentially methylated CpG sites (Fig. 1; Additional file 1: Table S2). The *TP63* gene was identified as a central hub within this specific cluster of co-expressed genes and is henceforth referred to as the TP63 cluster.

**Fig. 2:**
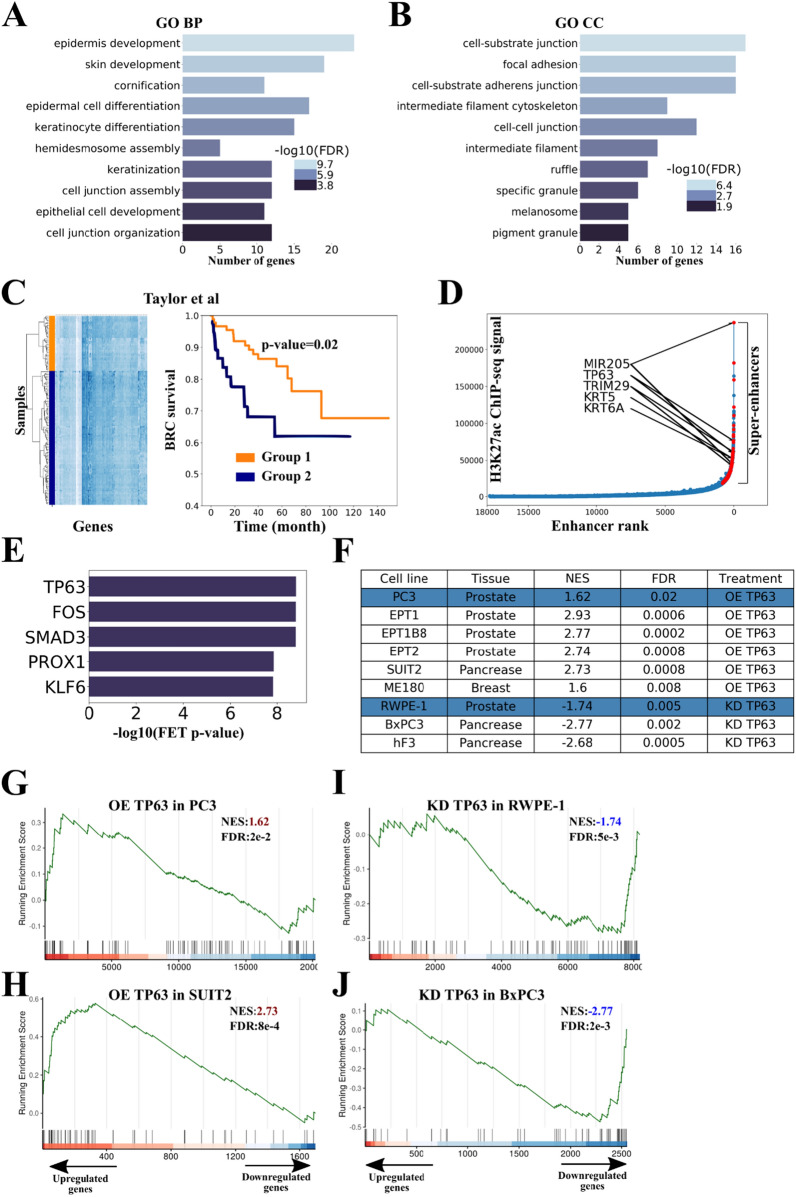
TP63 regulates the cluster of genes associated with epigenetic variability and genome instability in PRAD. **A**, **B** Gene Ontology (GO) analysis of the cluster genes. GO terms related to biological process (**A**), and cellular components (**B**) are shown. FDR is indicated with a color scale. **C** (Left panel) Hierarchical clustering of samples from the Taylor dataset [3] based on the expression of cluster genes revealed two distinct groups characterized by low (orange, group 1) and high (blue, group 2) mean expression of genes of TP63 cluster. The heatmap displays the expression levels of TP63 cluster genes. (Right panel) Biochemical recurrence-free (BRC) survival curves of the two patient groups (corresponding to hierarchical clustering of samples from the Taylor dataset, group 1 and group 2). The p value was calculated using the log-rank test. **D** Hockey stick plot based on input-normalized H3K27ac signals in PrEC cell line. Super-enhancer-associated cluster genes are highlighted with red. **E** Results of TF-enrichment analysis (CHEA3) for the cluster of interest. *FET* Fisher's exact test. **F** Table summarizing RNA-seq data evaluating the cluster genes signature upon *TP63* overexpression (OE) or knockdown (KD) in the indicated cell lines. Normalized enrichment score (NES) was calculated using the GSEA function from clusterProfiler package [29]. Blue rows indicate data from this study. **G**, **H** Gene set enrichment analysis (GSEA) plots evaluating the cluster genes signature upon *TP63* overexpression (OE) in PC3 (PRAD, **G**) and SUIT2 (GSE115462, pancreatic cancer, **H**). *NES* Normalized enrichment scores. **I**, **J** GSEA plots evaluating the cluster genes signature upon *TP63* knockdown (KD) in RWPE-1 (prostate basal epithelium, **I**) and BxPC3 (GSE115462, pancreatic cancer, **J**)

Based on GSEA, the group of genes downregulated in PRAD was significantly enriched with genes of this cluster (Additional file 1: Figure S1A). Out of 1645 CpG sites associated with the cluster, 1539 sites (94%) were hypermethylated. GO term analysis revealed genes that were enriched for the “epidermis development,” "epithelial cell development," and "focal adhesion processes" (Additional file 1: Table S2; Figs. 2A, B).

To determine the clinical relevance of the identified gene cluster, we analyzed the expression data of the TP63 cluster’s genes in biopsy samples of prostate tumors from the Taylor et al. series (GSE21032). Hierarchical clustering of samples based on the expression of TP63 cluster genes revealed two distinct groups of patients characterized by low (orange, group 1) and high (blue, group 2) mean expression of TP63 cluster genes. We found that the patients from group 1 were significantly associated with late biochemical recurrence (*p* = 0.02, Fig. 2C). [3]. We found that the cluster exhibited significant enrichment with signatures of basal prostate epithelium, but not luminal epithelium or stromal cells (Fisher exact test OR = 50, *p* = 3e-11) [22]. In addition, we confirmed that the cluster is regulated by super-enhancers (SEs) specific to the prostate basal epithelium (43 genes, OR = 3, *p* = 1.5e-5, Fig. 2D). This finding may suggest its association with cell lineage specification and cell fate decision functions [23].

Often, clusters of co-expressed genes are regulated by only one or few transcription factors. We used the CHEA3 program [24] and identified the top five most likely candidates regulating the cluster: TP63, FOS, SMAD3, PROX1, KLF6 (Fig. 2E). Since only one of listed TF (TP63) belongs to the cluster of co-expressed genes, we hypothesized that it could serve as a probable regulator of the cluster. TP63 is a recognized master regulator of epithelium development [25–28], and its expression is considerably decreased in PRAD and absent in all studied PRAD cell lines (Additional file 1: Figure S1B and C). To uncover the effects of TP63 on the cluster, we overexpressed *TP63* in the PC3 PRAD cell line (similar to most PRAD cell lines, PC3 cells lack endogenous expression of *TP63*) and knocked down *TP63* in RWPE-1 normal prostate epithelial cells (Additional file 1: Figure S1D) and performed RNA-seq analysis. Expression of *TP63* led to a considerable increase in the expression of the cluster genes in PC3 cells (Figs. 2F and G). On the contrary, knockdown of *TP63* resulted in a significant decrease in the cluster gene expression in RWPE-1 cells (Figs. 2F and H). We proposed that the association between *TP63* and the cluster gene expression could be common across different cancer types. Through this process, we demonstrated that expression of the cluster genes depended on *TP63* in pancreatic cancer (SUIT2, BxPC3, hF3; Figs. 2F-H, Additional file 1: Table S1), prostate cancer (EPT1, EPT1B8, EPT2; Fig. 2F, I and J, Additional file 1: Table S1), and cervical cancer (ME180; Fig. 2F, Additional file 1: Table S1) cell lines. To show that *TP63* directly regulates the cluster genes, we performed ChIP-seq with anti-TP63 antibodies using RWPE-1 normal prostate basal epithelial cell line and PC3 PRAD cell line with overexpression of *TP63* (PC3-TP63). In both normal basal epithelium and PC3-TP63 cells, the cluster genes were significantly enriched with ChIP-seq peaks of TP63 (Fisher exact test OR = 5.6 and OR = 5.7, *p* = 6.3e-17 and *p* = 7.8e-21, respectively). Therefore, we confirmed that transcription of the cluster is regulated by TP63.

### CpG sites associated with the TP63 cluster belong to TP63-dependent enhancers and super-enhancers

**Fig. 3:**
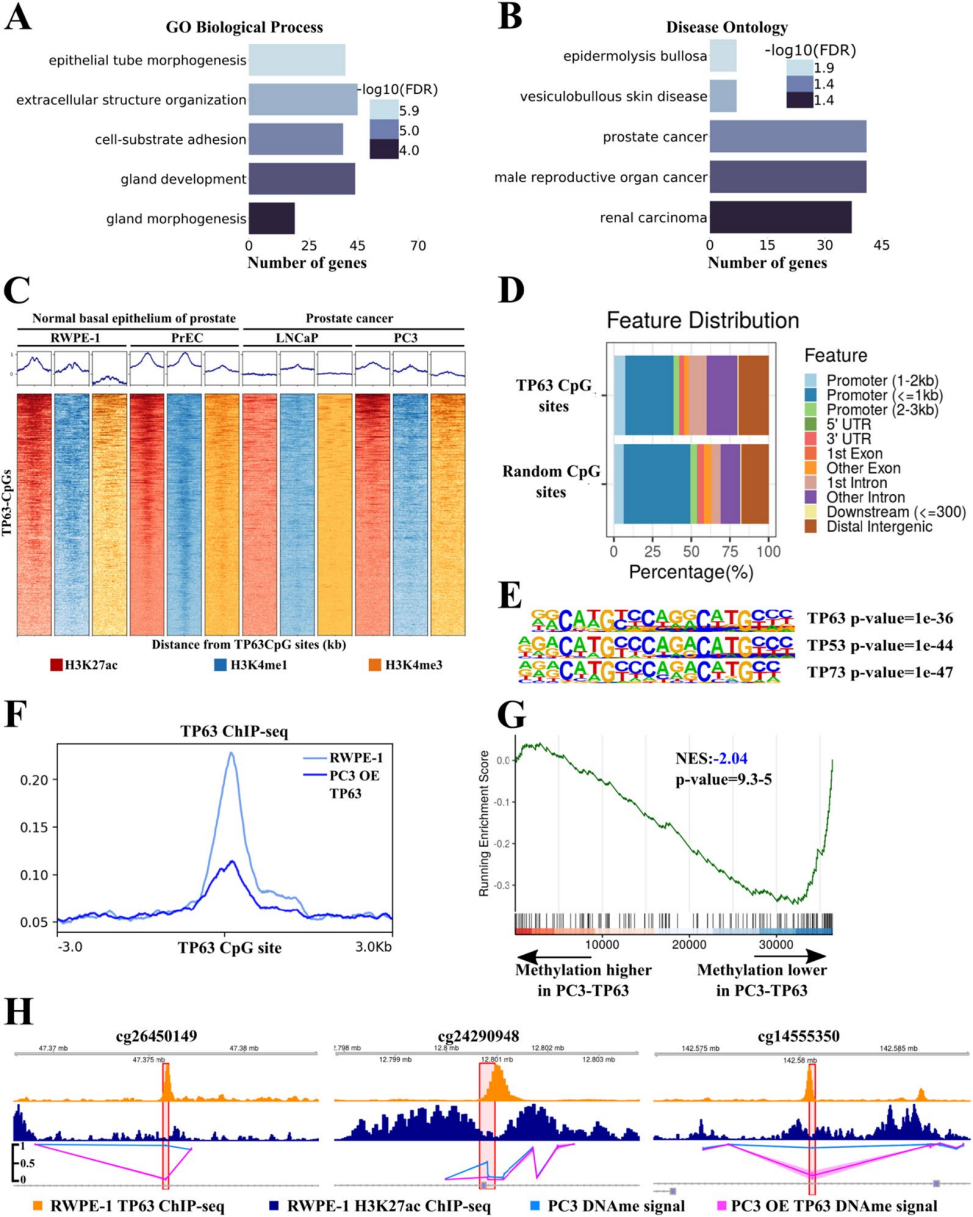
CpG sites associated with TP63 cluster lie in TP63-dependent enhancers and super-enhancers. **A**, **B** Gene Ontology (GO) analysis of genes located close to TP63 CpG sites. GO terms related to biological process (**A**) and disease ontology (**B**) are shown. FDR is indicated with a color scale. **C** ChIP-seq density plots of H3K27ac, H3K4me1, and H3K4me3 enrichments at TP63 CpG sites in the RWPE-1, PrEC, LNCaP, and PC3 cell lines. Each column represents a 6-kb interval centered on a CpG site. **D** Bar plot of genomic distribution of the TP63 CpG sites (top) and random CpG sites (bottom). **E** Representation of p53 protein family motifs enriched at TP63 CpG sites using HOMER. **F** Mean TP63 ChIP-seq signal at TP63 CpG sites in the RWPE-1 cell line and PC3 with overexpression (OE) of *TP63*. **G** GSEA-like plot for methylation data evaluating the TP63 CpG sites signature upon *TP63* overexpression in PC3. **H** ChIP-seq profiles of H3K27ac (top track) and TP63 (middle track) in RWPE-1 cell line and DNA methylation profile in PC3 cell line (bottom track) following overexpression (OE) of *TP63* or GFP as a control at three CpG sites

Here, we proposed that transcription of the TP63 cluster is associated with changes in methylation of 1645 CpG sites (TP63 CpG sites from now on, Additional file 1: Table S3). Based on this assumption, we performed GO analysis of genes located in proximity to these CpG sites. Our subsequent analysis confirmed their association with prostate development, morphogenesis, and cancer (Figs. 3A, B, Additional file 1: Table S3). To determine the functional role of these sites, we used open ChIP-seq data on histone modifications H3K27ac, H3K4me1, and H3K4me3 for normal prostate basal epithelium (RWPE1, PrEC; Additional file 1: Table S1) and PRAD (LNCaP, PC3; Additional file 1: Table S1) cell lines. TP63 CpG sites were found to be enriched with enhancer features in PrEC and RWPE-1 cell lines, but not in PRAD cells. They were localized mainly in distal (non-promoter) regions compared to random CpG sites (Figs. 3C, D). Using the activity-by-contact (ABC) models [30], we predicted enhancers for the PrEC normal basal epithelium cell line. Out of 1645 TP63 CpG sites associated with the cluster, 175 CpG sites resided in the ABC enhancer region (Fisher exact test OR = 3.8, *p* = 2e-45). Moreover, TP63 CpG sites were considerably enriched with SEs of the PrEC cells line (Fisher exact test OR = 5.1, *p* = 2e-63). Therefore, we found that TP63 CpG sites belonged to enhancers specific for prostate basal epithelium. A survey for transcription factor motifs within 100 bp from TP63 CpG sites showed significant enrichment of motifs for the binding of the p53 family of transcription factors (Fig. 3E). In addition, TP63 CpG sites showed intensive TP63 ChIP-seq signals in the RWPE-1 and PC3-TP63 cell lines (Fig. 3F). Therefore, these data confirmed that TP63 CpG sites are located in TP63-dependent enhancers of basal epithelium.

Having established a link between TP63 and TP63 CpG sites, we evaluated the functional role of TP63 in regulating methylation of these CpG sites next. We performed whole-genome methylation analysis using the Illumina MethylationEPIC chip in the PC3 cell line with *TP63* overexpression. TP63 CpG sites were almost completely methylated in PC3 (Additional file 1: Figure S2A). Upon induced expression of *TP63* in PC3, the level of TP63 CpG sites methylation significantly decreased (Fig. 3G). CpG sites with decreased methylation level under TP63 overexpression belonged to TP63 ChIP-seq peaks in RWPE-1 and PC3-TP63 cell line (Fig. 3H). To show that a decrease in the expression of *TP63* can lead to increased methylation of TP63 CpG sites, we chose three CpG sites that alter the levels of methylation in overexpressed *TP63* in PC3 and belong to TP63 ChIP-seq peaks in the RWPE-1 cell lines. We performed a knockdown of the *TP63* gene in the RWPE-1 cell line and used MSRE-qPCR to show that methylation significantly increased in two of the three CpG sites (Additional file 1: Figure S2B and C). Thus, both increase and decrease of TP63 expression can lead to changes in methylation of TP63 CpG sites.

Collectively, these results strongly indicate that CpG sites associated with the TP63 cluster belong to TP63-dependent enhancers of prostate basal epithelium and their methylation level depends on TP63 expression.

### TRIM29 interacts with TP63 and regulates the expression of the TP63 cluster

**Fig. 4:**
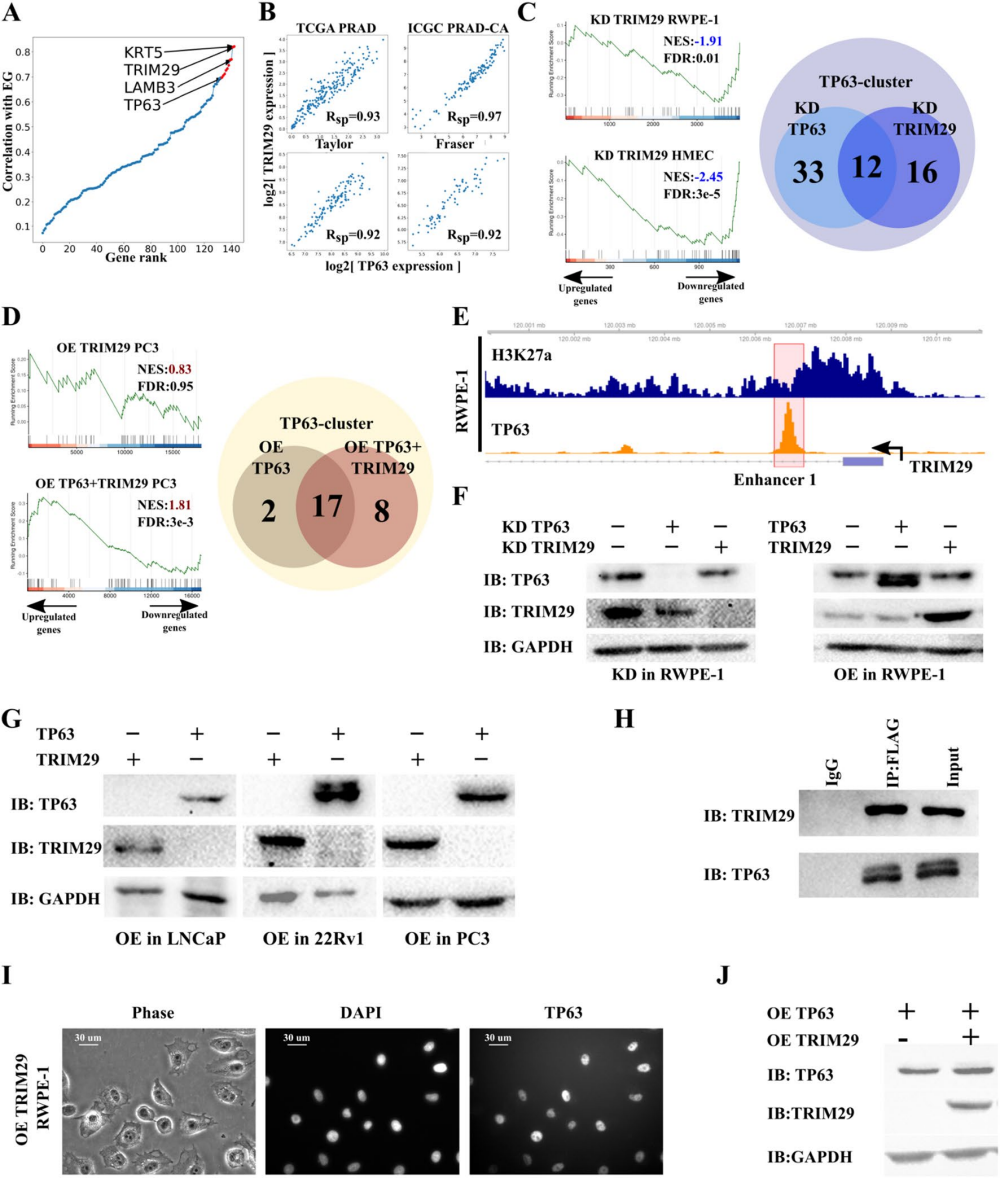
TRIM29 interacts with TP63 and regulates the expression of the TP63 cluster. **A** Eigengene of TP63 cluster significantly correlates with the expression of *TRIM29*. **B** *TP63* expression significantly correlates with the expression of *TRIM29* in four PRAD datasets. **C** Comparison of *TP63* and *TRIM29* knockdowns (KDs). GSEA plots evaluating the TP63 cluster signature upon *TRIM29* knockdown in RWPE-1 (prostate basal epithelium, left-top) and HMEC (GSE71375, basal breast epithelium, left-bottom). Venn diagram showing the overlap of differentially expressed TP63 cluster genes upon *TP63* and *TRIM29* knockdown in the RWPE-1 cells (right). *NES* Normalized enrichment scores. **D** Comparison of *TP63* and *TP63* + *TRIM29* overexpression (OEs). GSEA plots evaluating the TP63 cluster signature upon *TRIM29* overexpression in PC3 (PRAD, left-top) and *TP63* and *TRIM29* simultaneous overexpression in PC3 (PRAD, left-bottom). Venn diagram showing the overlap of differentially expressed TP63 cluster genes upon *TP63* overexpression and *TP63* and *TRIM29* simultaneous overexpression in PC3 cells (right). **E** ChIP-seq profiles of H3K27ac (top track) and TP63 (bottom track) in the RWPE-1 cell line of the *TRIM29* gene. Enhancer 1 of *TRIM29* in a red polygon. **F** Immunoblot (IB) analysis of knockdown (KD) of *TP63* and *TRIM29* (left) and *TP63* and *TRIM29* overexpression (OE, right) in the RWPE-1 cells. **G** Immunoblot (IB) analysis of overexpression (OE) of *TP63* and *TRIM29* in PRAD cell lines: LNCaP, 22Rv1, and PC3. **H** Interaction between TRIM29 and TP63 was detected by co-immunoprecipitation (co-IP) in the PC3 cells overexpressing *TRIM29-FLAG* and *TP63*. **I** Overexpression (OE) of *TRIM29* does not lead to the relocation of TP63 from the nucleus to the cytoplasm. Immunocytochemistry staining of TP63 in the RWPE-1 cells overexpressing *TRIM29*. **J** Immunoblot (IB) analysis of overexpression (OE) of *TP63* and *TRIM29* simultaneously in PC3 cells

Oncogenic or tumor suppressor properties of TP63 often depend on its protein partners in the cell [31]. In squamous cell carcinoma (SCC), TP63 is an oncogene, and interacts with pioneer transcription factors as SOX2 and KLF5 [26, 27]. By contrast, TP63 is a tumor suppressor in PRAD and cervical cancer [32]. Since the function of TP63 partners still remains relatively unknown in these types of cancer, we decided to identify potential TP63 co-regulators in the TP63 cluster. According to Langfelder and Horvath, the eigengene of the cluster of co-expressed genes (pattern of cluster expression) has the highest correlation with the expression rate of potential regulators of the co-expressed gene cluster in WGCNA [16]. *KRT5* and *TRIM29* are the top two genes in terms of degree of expression correlation with TP63 cluster eigengene expression (Fig. 4A). KRT5 is a cytokeratin marker of basal epithelium. TRIM29 is a ubiquitin ligase of the TRIM family which is a known partner of TP53 (a TP63 homologue) and regulates its transcription factor activity [33]. Therefore, TRIM29 can be an important regulator of the TP63 cluster. Analysis of four PRAD RNA-seq datasets [3, 4, 34, 35] (Additional file 1: Table S1) showed a strong correlation between *TRIM29* and *TP63* (Fig. 4B). This observation concurs with TRIM29 regulating the basal invasive program via TP63 in bladder cancer [36]. Thus, we aimed to decipher the effect TRIM29 has on the TP63 cluster in PRAD-related cell lines.

To this end, we performed *TRIM29* knockdown in a prostate basal epithelium cell line RWPE-1 and induced expression of *TRIM29* in PC3 cell line followed by RNA-seq analysis. *TRIM29* knockdown in RWPE-1 led to a significant decrease in the expression of the TP63 cluster genes (Fig. 4C), which agrees with previous observations from the dataset on *TRIM29* knockdown in normal breast epithelial cell lines HMEC and MCF10A (Additional file 1: Table S1, Fig. 4C, and Additional file 1: Figure S3A). Interestingly, knockdown of *TP63* and *TRIM29* in RWPE-1 resulted in changes in the expression of overlapping gene sets of the cluster (Fig. 4C) involved in processes such as epidermis development and differentiation, keratinocyte differentiation, and cell junction organization (Additional file 1: Table S4). On the other hand, overexpression of *TRIM29* in PC3 did not affect the cluster gene expression. Yet, combined overexpression of *TP63* and *TRIM29* increased the number of differentially expressed genes of the TP63 cluster (Fig. 4D).

Since TP63 and TRIM29 regulate the TP63 cluster simultaneously, we inquired whether TP63 and TRIM29 can mutually regulate each other. We proposed three possible ways of mutual regulation of the expression of these genes. (1) TP63 or TRIM29 can directly regulate the expression of each other. (2) TRIM29 could change the localization of TP63 on the cell, and (3) TRIM29 could act as ubiquitin-ligase, leading to degradation of TP63. Even though TP63 binds with TRIM29 enhancer in prostate basal epithelium and upon *TP63* overexpression in PC3 (Fig. 4E, Additional file 1: Figure S3B), knockdown of *TP63* in PRAD cell lines did not lead to an increased TRIM29 expression (Fig. 4G–J). Overexpression or knockdown of *TRIM29* did not change the expression of TP63 (Figs. 4G–J). Thus, in PRAD, TP63 and TRIM29 did not regulate each other at the transcription level.

We then postulated if TRIM29 can interact with TP63 and thus regulate its transcription factor activity. We induced overexpression of TRIM29-FLAG and TP63 in a PRAD cell line PC3 followed by immunoprecipitation with FLAG. Results showed that TP63 and TRIM29 interact with each other (Fig. 4H). Furthermore, the mass spectrometry analysis of TRIM29 partner proteins confirmed the interaction between TRIM29 and TP63 in RWPE-1 (Additional file 1: Table S5). To identify the region of TRIM29 necessary for interaction with TP63 protein, we generated PC3 cells stably expressing the series of truncated mutants of TRIM29-FLAG and full-sized TP63. WB analysis showed that TP63 co-purified only with full-sized TRIM29. To determine the domain of TP63 necessary for interaction with TRIM29 we created constructs coding truncated forms of TP63 FLAG tagged. Full-length TP63 or TP63-truncated were co-transfected with TRIM29 into HEK293 cells. WB analysis revealed that TRIM29 co-purify with all truncated forms of TP63 (Additional file 1: Figure S4). Previous studies have shown that TRIM29 can lead to proteasomal degradation or change of localization of proteins [37–39]. TRIM29 can translocate TP53—homologue of TP63 from the nucleus to cytoplasm [33]. We hypothesize that TRIM29 might regulate TP63 activity by a similar mechanism. Hence, overexpression of TRIM29 in RWPE-1 did not lead to translocation of TP63 from the nucleus to cytoplasm (Fig. 4I). Also, overexpression of *TRIM29* did not lead to degradation of TP63 (Figs. 4F and J), as in the case of the STING protein [37]. Consequently, TRIM29 does not regulate TP63 cluster expression by modulating TP63 abundance in the nucleus. All these data indicate that TRIM29 can modulate TP63 transcription factor activity and thus regulate cluster expression. It remains unclear, though, how the TP63 activity is regulated. Further research will need to be investigated to determine how TP63 is regulated.

### Restoration of TRIM29 expression promotes the decrease of chromosomal instability in PRAD

**Fig. 5:**
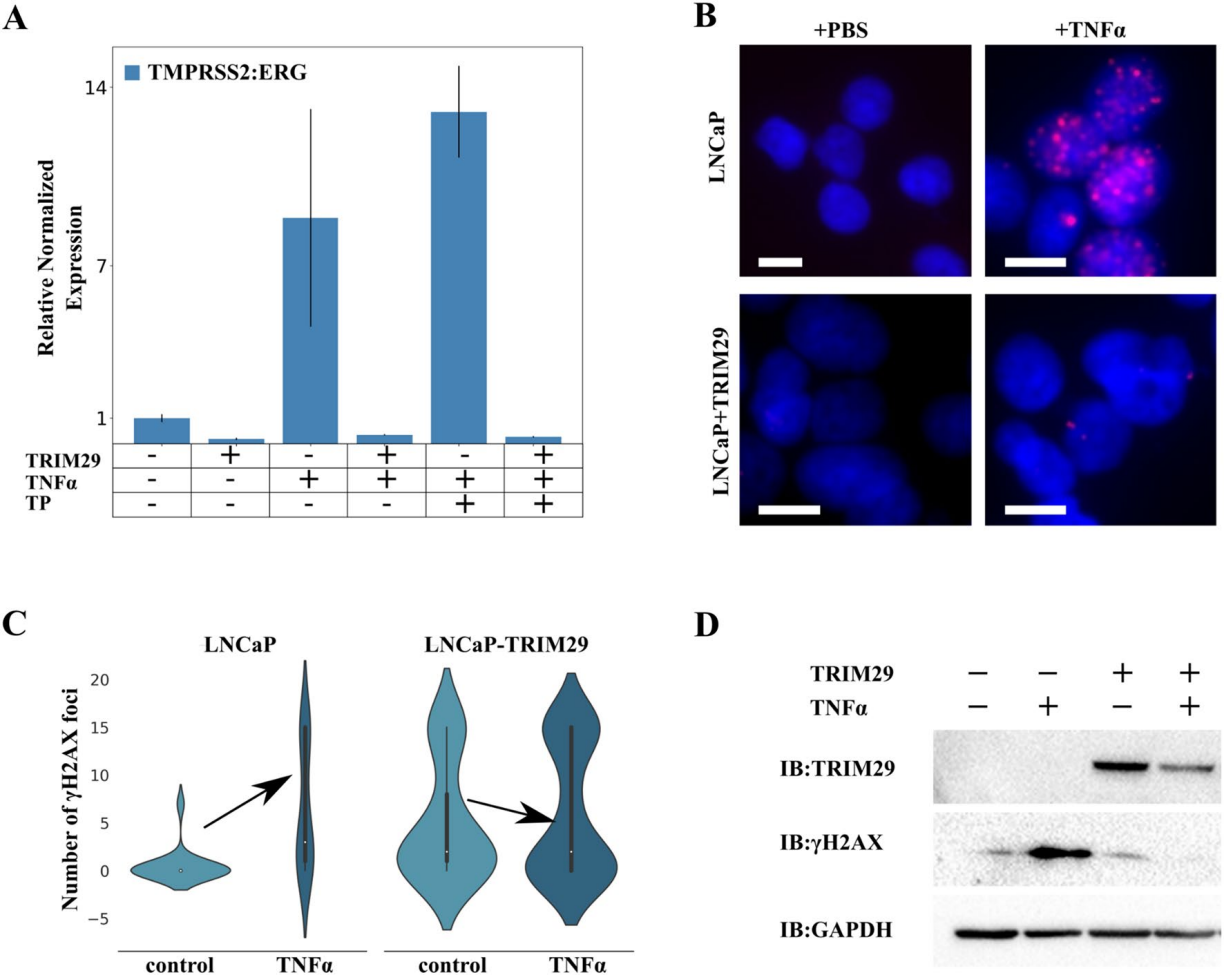
Restoration of TRIM29 expression promotes decrease in genome instability in PRAD. **A** qRT-PCR analysis of *TMPRSS2:ERG* fusion transcripts from the LNCaP cells with the indicated treatments for 48 h. *TP* testosterone propionate. **B** γH2AX foci formation upon treatment of LNCaP and LNCaP-TRIM29 cells with TNFα (100 ng/mL). Cells were processed 48 h post-treatment. Scale bar indicates 25 μm. **C** Violin plots of the γH2AX foci number per nucleus in LNCaP and LNCaP-TRIM29 cells before and after TNFα (100 ng/mL) treatment. **D** Immunoblot (IB) analysis of lysates from LNCaP and LNCaP with stably expressed TRIM29 cells stimulated with TNFα (100 ng/mL)

Earlier, we demonstrated that the TP63 cluster is associated with increased chromosomal instability index and is not enriched with DNA repair proteins family of genes. We investigated two datasets that contained whole-genome screening of 28 genotoxic agents in retinal pigment epithelium [40] and two agents in HeLa cells [41]. Interestingly, there was no significant association between the TP63 cluster and the response to genotoxic stress (Fisher exact test, Additional file 1: Table S5), which led us to hypothesize that the association between TP63 cluster with chromosomal instability can be a consequence of the association of a cluster's hub gene with chromosomal instability. Upon literature search, as expected, the most probable candidate for chromosomal instability regulator was TRIM29. Previously, TRIM29 was found to be a scaffold protein of DNA double-strand break repair system in HeLa (cervical cancer) [42]. Also, TRIM29 exhibited radioprotective function in a number of studies [33, 42, 43]. Moreover, TRIM29 is a regulator of the TP63 cluster. Considering this, we decided to examine the effect of TRIM29 on chromosomal instability in PRAD. We first performed immunoprecipitation of TRIM29 from the nuclear fraction of RWPE-1 followed by Mass spectrometry analysis of the precipitate. Data showed that TRIM29 binds to DNA repair proteins that belong to mismatch excision repair, nucleotide excision repair, and homologous recombination families, as well as TP53BP1 (Table 1: Table S5). Therefore, a low abundance of TRIM29 can promote impairment in repairing double-strand breaks, and consequently, the accumulation of chromosomal instability. Then we studied the formation of *TMPRSS2:ERG* fusion genes, a gene fusion that occurs in more than half of PRAD cases, as a model of chromosomal instability [44]. As demonstrated previously, inflammation-related stress (effect of TNFα) promotes accumulation of double-strand DNA breaks in PRAD and accumulation of fusions between the *TMPRSS2* gene and the ETV family of proteins: *TMPRSS2:ERG* and *TMPRSS2:ETV* [45]. We showed that TRIM29 (LNCaP-TRIM29) overexpression in an LNCaP cell line resulted in a sixfold decrease in the frequency of *TMPRSS2:ERG* fusions compared to the parental cell line. The effect of TNFα did not lead to considerable growth of fusion rate in LNCaP-TRIM29 but resulted in a ninefold increase in fusion rate in the LNCaP line (Fig. 5A). Since fusion formation is mediated by androgens [45], we hypothesized that the addition of TNFα together with testosterone to LNCaP would increase the fusion rate. Indeed, when added with TNFα, testosterone propionate caused a 13-fold increase in gene fusion rate in LNCaP but did not influence fusion rate in LNCaP-TRIM29 (Fig. 5A). We demonstrated that overexpression of TRIM29 in LNCaP cells led to a decrease in the number of γH2AX foci, which are markers of double-strand breaks in DNA, in response to TNFα (Anova p-value < 0.05; Figs. 5B–D). In addition, we showed that knockdown of TRIM29 in RWPE-1 cells resulted in a significant increase in the number of γH2AX foci (*t* test *p*< 1e-8, Additional file 1: Figure S5). Overall, our findings provide evidence for the protective role of TRIM29 against DNA damage induced by inflammatory stress in the cell lines studied.

**Table 1:** TRIM29-interacting proteins in RWPE-1 identified by mass spectrometry

Gene Name	# of spectral counts IP-TRIM29		# of spectral counts IP- IgG		Protein family
MSH2	7	15	1	0	MMR
MSH6	6	14	5	3	MMR
RAD23B	3	1	0	0	NER
CENT2	4	3	0	0	NER
DDB1	8	13	3	0	NER
CHAF1A	1	3	0	0	Other
PRPF19	15	14	3	3	Other
TP53BP1	5	2	0	0	Other

*MMR* mismatch excision repair, *NER* nucleotide excision repair, *Other* other identified genes with known or suspected DNA repair function

## Discussion

In this study, we conducted an integrative analysis of RNA-seq, DNA methylation, and copy number variation data of PRAD TCGA. Our analysis revealed a cluster of 143 co-expressed genes that are associated with epigenomic variability and chromosomal instability. This cluster is enriched with gene signatures of normal basal epithelial cells and is involved in biological processes such as epithelium development and differentiation. We also demonstrated that TP63 and TRIM29 regulate expression of this cluster. TP63 is a transcription factor that acts as a master regulator of epithelium development. It regulates the expression of basal markers such as *KRT5/6*, *KRT14*, *S100A2*, and *miR-205* [46]. In many squamous-like cancers (pancreas, stomach, head, and neck cancers), *TP63* is an oncogene, with high expression linked to a poor prognosis. However, in PRAD [25, 26, 32, 46], high *TP63* expression is associated with a more positive mortality outcome and tumor suppressor properties. TRIM29 (or ATDC) is a member of the TRIM family and functions as a ubiquitin ligase. Similar to TP63, TRIM29 has both oncogene and tumor suppressor properties depending on the cancer type [47]. In PRAD, *TRIM29* expression is considerably lower than in normal tissue [48]. TRIM29 has been shown to regulate the expression of basal program genes (*KRT5, KRT14)* through TP63 [36] in bladder and cervical cancers. We have shown that, in PRAD, TP63 binds to the *TRIM29* enhancer region but does not regulate TRIM29 expression, unlike bladder cancer. Interestingly, knockdown of either *TP63* or *TRIM29* leads to a decrease in the expression of the TP63 cluster genes. However, overexpression of *TRIM29* does not influence the expression of the *TP63* gene cluster. Simultaneous overexpression of *TP63* and *TRIM29*, however, results in greater expression than *TP63* overexpression alone. Therefore, our study reveals that TRIM29 acts as a modulator of the TP63 transcription program in PRAD.

Furthermore, we have shown that TP63 and TRIM29 form a complex in the prostate basal epithelium. TRIM29 does not promote TP63 degradation or translocation from the nucleus. Thus, we speculate that TRIM29 can post-translationally modify TP63 owing to its ubiquitin-ligase activity [37], or susceptible sumoylase activity [49], can post-translationally modify TP63. An ubiquitin-ligase WWP1 influences TP63 transcription factor activity and stability [50]. Simultaneous expression of TP63 and WWP1 increases the expression of genes KRT14 and KRT5 [50], which concurs with our data from PC3 and bladder cancer cell lines overexpressing TP63 and TRIM29 genes. Moreover, monoubiquitination of the DNA-binding domain of TP53, a TP63 homologue, has been shown to modify TP53 affinity to DNA [51]. Hypothetically, this can work for TP63 as well due to the high homology of DNA-binding domains of TP63 and TP53. In addition, in some cases, ubiquitination of TP63 has been shown to require sumoylation [52]. The sumoylation site is located close to the SAM domain responsible for protein–protein interactions of TP63. Therefore, the sumoylation of TP63 can plausibly affect the choice of protein partners and thus influence its transcription factor activity. Hence, our hypothesis on TRIM29-mediated regulation of TP63-dependent genes is compelling and provides groundwork for further study of TRIM29's role in post-translational regulation of TP63 activity.

TP63 is a pioneer transcription factor [53], that establishes the epigenetic landscape of epidermal enhancers and super-enhancers in squamous-like types of cancer [25–28]. Here, for the first time, we have demonstrated that TP63 can regulate the level of methylation of enhancers specifically in basal epithelium cells. However, not all the CpG sites associated with the TP63 cluster are subject to demethylation upon expression of TP63 [25, 26]. This result can potentially be a consequence of increased hydroxymethylation of cytosines (hmC) at some enhancers by TP63 [54]. Therefore, further research will be needed to investigate the regulation of hmC levels in enhancers of basal epithelium by TP63. In addition, the level of methylation of TP63-dependent CpG sites can be a potential biomarker of TP63 and TRIM29 levels. If so, TP63 and TRIM29 levels can be indirectly evaluated by quantification of CpG methylation in extracellular DNA from urine or blood plasma [55].

We have demonstrated that the TP63 cluster is associated with chromosomal instability, although a direct link between the cluster and chromosomal instability is doubtful since the cluster does not contain any genes involved in the DNA repair system. Furthermore, the cluster genes are not associated with decreased survival upon exposure to DNA-damaging agents [40, 41]. Thus, we hypothesize that a single hub gene of the cluster may be associated with chromosomal instability. Previous studies have shown TRIM29's role as a scaffold protein in the DNA double-strand break repair system [42]. Notably, DNA repair system proteins MSH2/6 and RAD50 are protein partners of TRIM29, which agrees with Masuda's findings for the HeLa cell line [42]. Therefore, decreased expression of TRIM29 in PRAD and other cancer types may impair DNA repair and increase chromosomal instability. Our study confirms this hypothesis using the model of *TMPRSS2:ERG* fusion gene formation in PRAD. Earlier work has shown that the formation of the fusion occurs via the androgen-dependent pathway and is induced by oxidative or inflammatory stress [45]. We showed that overexpression of *TRIM29* significantly decreases the rates of *TMPRSS2:ERG* fusion formation even under inflammatory stress. Moreover, overexpression of *TRIM29* significantly reduces the number of γH2AX foci under the TNFα treatment. Our results are consistent with previous studies showing that overexpression of TRIM29 decreases the amount of γH2AX after exposure to ionizing radiation [42]. Although TRIM29 is not expressed in PRAD cancer cells, we speculate that the loss of TRIM29 during basal cell differentiation may lead to decreased resistance to inflammatory and oxidative stress in luminal cells. This process could potentially result in the formation of *TMPRSS2:ERG* fusion and oncogenic transformation. It is possible that this is a natural process, as age and inflammatory stress are known risk factors for prostate cancer. In addition, the tumor cell secretome may decrease TRIM29 expression in the basal epithelium, resulting in a population of cells with reduced chromosomal stability. Therefore, our study provides important insights linking decreased *TRIM29* expression to increased accumulation of chromosomal instability.

## Conclusions

We have identified a new partner protein for TP63, TRIM29 ubiquitin ligase, which directly regulates the activity of TP63 as a transcription factor. We have shown TP63- and TRIM29-dependent regulation of basal enhancer methylation and chromosomal instability in PRAD. Our findings provide a solid rationale for studying the role of TRIM29 in the regulation of basal epithelium development via TP63 regulation in the prostate gland and epithelium in general.

## Materials and methods

### Data collection

#### The Cancer Genome Atlas (TCGA) PRAD

Due to the high heterogeneity of PRAD data, for the analysis, we selected only samples of patients that meet the following criteria: Gleason scores 6 through 8; no hormonal therapy; over 55-year old (Additional file 1: Table S1). Also, we did not include samples marked as contaminated with admixtures of other tissues in TCGA. After quality control and filtration steps, 225 samples of cancerous and 52 normal tissue samples remained.

### Processing of RNA-seq data

#### TCGA and ICGC data

Level 3 data with mapped readings were used. To build correlation networks, TPM normalization was applied. Only those genes that were expressed in over 50% cancer samples (> 1TPM) with expression variability lying above the 25% percentile were included in further analysis. For differential analysis between cancer and not cancer, DESeq2 package was used. Genes were considered differentially expressed, if abs(logFC) > 0.5 and FDR < 0.05.

#### Taylor and fraser data

Normalized intensities were downloaded with GEO using GEOquery.

### Data on knockdown and overexpression of TP63 and TRIM29

#### RNA-seq data

Pre-processing of raw readings was performed with trimmomatic. Then, the reads were mapped onto the genome version hg19 using STAR software. To quantify gene coverage, the featureCounts software with gencode v37 annotation was used. For differential analysis, the DESeq2 package was used. Genes were considered differentially expressed, if |logFC|> 0.5 and FDR < 0.05.

#### Hybridization chip data

Normalized intensities were downloaded with GEO using GEOquery. Differential analysis of expression was performed with the Limma package.

### Analysis of DNA methylation data

#### TCGA data

##### The 450 K Methylation platform

Files containing raw intensity values (*.idat files) were downloaded from the TCGA portal. To process the data, RnBeads [56] program with the following setup parameters was used: methylumi.noob [57] + BMIQ [58] normalization; limma algorithm for differential analysis. CpG site was considered differentially methylated if |Δβ| > 0.2 and FDR < 0.05.

#### Data on TP63 overexpression in PC3

##### EPIC Methylation platform

To process the data, RnBeads [56] program was used with the following setup parameters: ENmix [59] + BMIQ [58]; limma algorithm for differential analysis [60]. To search for motifs of transcription factors, HOMERv4 [61] software with the motif database HOCOMOCOv11 [62] were used.

### Analysis of CNV data

Level 3 open data obtained on Affymetrix 6.0 platform were used. A region was considered to have an altered copy number if the absolute value of the ratio of intensities between sample and control exceeded 0.3. To describe chromosomal instability, chromosomal instability index (CIN), equal to the ratio of total length of CNV to the length of a chromosome, averaged across all chromosomes, was used.

### Determination of clusters of co-expressed genes associated with epigenomic variability and chromosomal instability

To generate clusters, we used the WGCNA package [16] with standard parameters. We found 13 clusters of co-expressed genes in TCGA data. For each cluster, we determined its expression pattern, cluster eigengene (EG), which is the main component of PCA.

As it has been demonstrated in a previous work, the level of methylation of CpG sites can strongly depend on the ratio of various types of prostate cells in a sample [21]. We used the MuSic package [63] and prostate scRNA-seq data [22] to calculate the ratio of stromal, basal, and luminal epithelium cells in the prostate gland in TCGA samples. Calculated ratios of different epithelial cells were used as co-variants for further regression analysis. To determine the set of non-overlapping CpG sites associated with each cluster, we used the following regression model:

$${{\text{EG}}}_{i} = {{\text{a}}}_{i} + {{\text{b}}}_{i}*{{\text{CpG}}}_{j} + {{\text{Cov}}}_{i} + {{\text{Err}}}_{i},$$

where EG_*i*_ is cluster *i* eigengene; a_*i*_, a constant; b_i_, coefficient by the vector of methylation of CpG_i_; and Cov, co-variants that include the ratio between various types of cells in a sample and the rest eigengenes of the cluster except for the studied one to determine only CpG sites uniquely associated with the cluster. We thus determined 14,443 differentially methylated CpG sites associated with eigengenes of the clusters.

We used linear regression to determine gene clusters associated with CIN:

$${\text{CIN}}\hspace{0.17em}=\hspace{0.17em}{{\text{a}}}_{i}\hspace{0.17em}+\hspace{0.17em}{{\text{b}}}_{i}*{{\text{EG}}}_{i}\hspace{0.17em}+\hspace{0.17em}{{\text{Cov}}}_{i}\hspace{0.17em}+\hspace{0.17em}{{\text{Err}}}_{j},$$

where CIN is chromosomal instability index, EG_i_, is cluster i eigengene; a_i_, a constant; b_i_, a coefficient at EG_i_; Cov, co-variants that include the ratio between various types of cells in a sample.

To determine association of clusters with differentially expressed genes between cancer and normal tissue, we used GSEA [20], where genes of each cluster were used as signatures.

### Prediction of super-enhancer-associated genes

We used the ROSE framework [23] with DNAse-seq and H3K27ac ChIP-seq for normal prostate epithelial cell line PrEC for SEs prediction. For the prediction link between genes and enhancers and SEs, we used the Activity-by-contact framework [30].

### Cell culture

Human RWPE-1 cells (ATCC^®^ CRL-11609^™^) were cultured in Keratinocyte Serum-Free Media (Gibco, USA). Prostate cancer cell lines (PC3, LNCaP, 22Rv1) were kindly provided by Dr. M. Lagarkova (Federal Research and Clinical Center of Physical–Chemical Medicine, Moscow, Russia) and were cultured in corresponding media supplemented with 10% FBS and penicillin/streptomycin. Human cells were grown at 37 ℃ and 5% CO_2_ in a conventional humidified CO_2_ incubator.

### Plasmids

The FLAG-tagged TRIM29 and its truncated forms were cloned into the LeGO-iG2 vector. pcDNA3-TP63-FLAG was obtained from addgene (cat. 26,979). FLAG-tagged TP63 was cloned штto LeGO-iT2. The truncated forms of TP63 were cloned into the pcDNA3.1 vector.

### Preparation of lentivirus particles

One day prior to transfection, Phoenix cells were inoculated onto 10-cm Petri dishes covered with 0.1% gelatin 7 × 10^5^ cells per dish. The cells were transfected with auxiliary plasmids containing the Rev (15.3% by mass to total DNA), RRE (29.8% by mass to total DNA), and VSV-F (5.6% by mass to total DNA) genes and target plasmid encoding TRIM29_FLAG. Transfection was performed using the TurboFect (ThermoFischer Scientific, US) transfection agent, 26.4 µL per 13.2 µg DNA. The procedure of transfection was performed in accordance with the manufacturer's recommendations. Virus-containing supernatant was collected 24, 48, and 72 h after transfection, filtered through a 0.45-µm filter and stored at – 70 ℃.

### Generation cell lines with stable expression of TP63 and TRIM29

Two days before transduction, cells were seeded into 24-well plate 2 × 10^4^ cells per well. One hour prior to transduction, 8 µg/mL polybrene was added. Cells were transduced with virus particles containing TP63-FLAG or TRIM29-FLAG. After 24 h, cell medium was replaced. Two days after transfection, cells were trypsinized and TP63/TRIM29-infected cells were isolated on a FACS BD Aria III cell sorter.

### Cell treatment with TNFα

Prior to treatment with TNFα, cells were cultured to 50–60% confluence layer. TNFα was added into the medium to a final concentration of 100 ng/mL and cultured in an incubator for 48 h.

### Chromatin immunoprecipitation

Chromatin immunoprecipitation was performed using the SimpleChIP Plus Enzymatic Chromatin IP Kit (Cell Signaling) according to the manufacturer's manual. In brief, 4 × 10^6^ cells were fixed with formaldehyde, cell nuclei were isolated, and chromatin was fragmented with micrococcal nuclease. Then, chromatin was incubated with 5 µL anti-TP63 antibodies (Cell Signaling #13109) overnight and TP63-bound DNA was precipitated on magnetic beads. The DNA was purified from bound protein complexes and used for massive parallel sequencing using the Illumina HiSeq-2500 or for quantitative PCR.

The following algorithm was used to analyze chromatin immunoprecipitation followed by sequencing: pre-processing of raw reads was performed by trimmomatic tool [64]; then the reads were mapped onto human genome version hg19 using the bwa mem [65] program with standard parameters. The *.bam files thus obtained were sorted and indexed with the samtools program. Reliably determined regions of TP63 or histone binding with determined modifications were found using the macs2 program [66]. ChIP-seq data were visualized using the deepTools program [67].

Paired-end libraries were prepared according to the manufacturer’s recommendations using NEBNext Ultra II DNA Library Prep Kit (New England Biolabs, USA). The libraries were indexed with NEBNext Multiplex Oligos kit for Illumina (96 Index Primers, New England Biolabs, USA). Size distribution for the libraries and their quality were assessed using a high-sensitivity DNA chip (Agilent Technologies). The libraries were subsequently quantified by Quant-iT DNA Assay Kit, High Sensitivity (Thermo Scientific, USA). DNA sequencing was performed on the HiSeq 2500 platform (Illumina, USA) according to the manufacturer’s recommendations, using the following reagent kits: HiSeq Rapid PE Cluster Kit v2, HiSeq Rapid SBS Kit v2 (200 cycles), HiSeq Rapid PE FlowCell v2 and a 1% PhiX spike-in control.

### TP63 and TRIM29 knockdown

TP63 and TRIM29 knockdown was performed using RNA interference (siTP63_1_F: CCGUGAGACUUAUGAAAUGTsT, siTP63_1_R: CAUUUCAuAAGUCUCACGGTsT; siTP63_2_F: UCACGACAGUCUUGUACAATsT, siTP63_2_R: UUGUACAAGACUGUCGUGATsT; siTRIM29_1_F: AUUGAUGAGCAAUUACUCUTsT, siTRIM29_1_R: AGAGUAAUUGCUCAUCAAUTsT; siTRIM29_2_F: ACCAAGUGAAGGUGAUCAUTsT, siTRIM29_2_R: AUGAUcACCUUcACUUGGUTsT). siRNA was transfected into RWPE-1 cells in a 24-well plate using HiPerFect Transfection Reagent (Qiagen, 301,704). siRNA, 68 ng, was mixed with 2 µL transfection reagent in 100 µL of Opti- MEM^™^ (Gibco) medium; the mixture was incubated at room temperature for 10 min. RWPE-1 cells were seeded onto a 24-well plate at 3.5 × 10^4^ cells/cm^2^. Cells were introduced into wells in 400 µL Keratinocyte SFM (Gibco) medium and the mixture of siRNA and transfection reagent was added to the cells immediately. Therefore, total medium volume was 500 µL in a well; final siRNA concentration, 10 nM. After 24 h, the medium was partially replaced with fresh Keratinocyte SFM (Gibco). The efficiency of transfection was evaluated 48 h after the addition of siRNA by western blotting.

### Cell lysate preparation for western blot analysis

Cell cultures were collected, and 1 × 10^6^ cells were counted by trypan blue exclusion and washed with ice cold PBS. Cells were then resuspended in 100 μL PBS and lysed with 100 μL of 2 × Laemmli Sample Buffer supplemented with β-mercaptoethanol by boiling for 5–10 min.

### Isolation of cytoplasm and nuclear fractions

Cells were washed with PBS and removed with a scraper in cool buffer A (0.35 M sucrose, 2 мM MgCl_2_, 0.1 mM EDTA, 0.1% Triton X-100, 10 mM Tris–HCl pH 8.0, 1 mM DTT, 0.1 mM PMSF, and protease inhibitors). Cell lysate was passed 10 times through a 21G needle syringe and a 23G syringe, 3 times. Then, it was centrifuged at 2000*g* for 10 min. The supernatant was collected as a cytoplasm fraction.

The precipitate containing nuclei was washed with 0.5 M sucrose and centrifuged at 10,000*g* for 10 min. The precipitate was resuspended in buffer C (400 mM NaCl, 20 mM HEPES pH 7.9, 25% glycerol, 1.5 mM MgCl_2_, 0.4 mM EDTA, 0.4 mM PMSF, 1 mM DTT, protease inhibitors) and passed 5 times through a 21G needle syringe. Then, it was incubated at + 4 ℃ for 40 min and centrifuged at 12,000*g* for 30 min. The supernatant was collected as the nuclear fraction.

### Immunoprecipitation

Cells were washed with PBS solution. Cell lysis buffer (Cell Signaling Technology, Great Britain) was added and incubated for 5 min on ice. Cells were removed with a scraper. Cell lysate was homogenized on an ultrasound bath. Lysate was centrifuged at 10,000*g* for 10 min. The supernatant was incubated with antibodies overnight at + 4 ℃ while stirring. Lysate with antibodies was incubated with magnetic beads for 1 h while stirring at room temperature. Magnetic beads were washed 5 times with a cell lysis buffer.

For western blotting, the proteins were eluted with Laemmli buffer at 95 ℃ for 5 min. Total protein concentration in the sample was determined using Bradford assay.

To prepare samples for mass spectrometry analysis, proteins were eluted with an elution buffer (8 M urea, 2 M thiourea, 10 mM Tris/HCl pH 8.0) on a shaker at 25 ℃ for 2 h.

### Western blotting

Electrophoretic separation of proteins was carried out in a Bio-Rad Mini Protean chamber. Then, semidry electrotransfer of proteins to a PVDF membrane was carried out in a Trans-Blot Turbo Transfer System (Bio-Rad, USA). The membrane was incubated in a blocking buffer (PBST, 5% nonfat dry milk). Then, it was incubated with primary antibodies against GAPDH (PAB932Hu02, Cloud-Clone Corp., USA), phospho-Histone H2A.X (Ser139) (JBW301, Sigma-Aldrich, USA), TP63 (D2K8X Cell Signaling Technology, USA) or TRIM29 (E1L4E, Cell Signaling Technology, USA) at+ 4 ℃ overnight, washed in PBST solution, and incubated with a solution of secondary antibodies conjugated with horseradish peroxidase (31460, Thermo Fisher Scientific, USA) for 1 h at room temperature. Proteins were visualized using Pierce ECL Western Blotting Substrate (Thermo Fisher Scientific, USA) at Chemidoc (Biorad, USA).

### Sample preparation for mass spectrometric analysis

After immunoprecipitation, DTT was added to the eluted samples to a final concentration of 5 mM and incubated for 30 min on a shaker at room temperature to restore disulfide bonds. Freshly prepared iodoacetamide was added to a final concentration of 10 mM and incubated in the dark for 20 min to alkylate the thiol groups of cysteine. The mixture was diluted with 35 mM ammonium carbonate solution fourfold. Sequencing Grade Modified Trypsin (Promega, USA) was added 0.1 μg trypsin per 10 μg protein and incubated on a shaker at 37 ℃ overnight. Trypsin was neutralized with a fivefold volume of a 5% formic acid solution. Desalting was performed using reversed phase chromatography on homemade StageTips with SDB-RPS filter according to the protocol from Rappsilber et al [68]. The samples were concentrated in a vacuum centrifuge and redissolved in 3% acetonitrile with 0.1% trifluoroacetic acid solution. The amount of protein in the sample was approximately 10 μg.

### LC–MS/MS analysis of tryptic peptides

After trypsinolysis, peptide fractions were loaded onto a column (diameter 75 μm, length 50 cm) with an Aeris Peptide XB-C18 2.6 μm sorbent (Phenomenex) in an aqueous solution containing 3% acetonitrile and 0.1% trifluoroacetic acid. Separation of peptides was performed on an Ultimate 3000 Nano LC System (Thermo Fisher Scientific), coupled to a Q Exactive HF mass spectrometer (Thermo Fisher Scientific) using a nanoelectrospray source (Thermo Fisher Scientific). Peptides were loaded onto a heated 40 ℃ column in buffer A (0.2% formic acid (FA) in water) and eluted with a linear (120 min) gradient 4 → 55% buffer B (0.1% FA, 19.9% water, 80% acetonitrile) in A at a flow rate of 350 nL/min. Before each new load, the column was washed with 95% buffer B in A for 5 min and equilibrated with buffer A for 5 min.

Mass spectrometry data were saved with automatic switching between MS1 scans and up to 15 MS/MS scans (topN method). The target value for the MS1 scan was set to 3 × 10^6^ in the range of 300−1200 *m*/*z* with a maximum ion injection time of 60 ms and a resolution of 60,000. The precursor ions were isolated with a window width of 1.4 *m*/*z* and a fixed first mass of 100,0 *m*/*z*. The precursor ions were fragmented by high-energy dissociation in a C-trap with a normalized collision energy of 28 eV. MS/MS scans were saved with a resolution of 15,000 at *m*/*z* 400 and at a value of 1 × 10^5^ for target ions in the range of 200−2000 *m*/*z* with a maximum ion injection time of 30 ms.

### Analysis of LC–MS/MS data

The conversion of the "raw" mass spectrometric data from the instrument into MGF (Mascot Generic Format) mass sheets was carried out using the ProteoWizard msconvert utility with the following parameters: MS Levels 2–2, Peak Picking 2–2, Threshold Peak Filter Absolute intensity—Most intense—1, Zero Samples 2–2.

For identification and quantitative analysis of protein partners, the MaxQuant program (v1.5.3.30) was used with the Andromeda algorithm against the protein database UniProt Knowledgebase (UniProtKB), the human taxon, with the following parameters: the accuracy of determining the parent and daughter ions was 20 and 50 ppm, respectively; protease, trypsin; one missed cleavage per peptide is possible; variable modification of methionine, oxidation; fixed modification of cysteine, carbamidomethylation. The reliability of identification of both peptides and proteins was limited to 1% FDR, which was determined using the "target decoy" approach. For quantitative label-free analysis, LFQ values were calculated using the MaxQuant software.

### Immunocytochemical analysis

Cells were washed with PBS solution 2 times for 5 min and fixed with 4% PFA for 30 min. The cells were washed 2 times for 5 min. Membranes were permeabilized with 0.1% Triton X-100 in PBS for 5 min. Nonspecific antigen adsorption was blocked by washing in 0.1% Tween 20 PBS solution 3 times for 5 min and then in a block solution (PBS, 0.1% Tween 20, 5% FBS, 5% goat serum) for 30 min. A solution of primary antibodies against GAPDH (PAB932Hu02, Cloud-Clone Corp., USA), phospho-Histone H2A.X (Ser139) (JBW301, Sigma-Aldrich, USA), TP63 (D2K8X Cell Signaling Technology, USA) or TRIM29 (E1L4E, Cell Signaling Technology, USA) was added in a block solution and incubated for 2 h at room temperature. The cells were washed from primary antibodies with a solution of 0.1% Tween 20 3 times for 5 min. A solution of secondary antibodies labeled with the Alexa Fluor 555 fluorophore was added in PBS and incubated in the dark for 1 h at room temperature. Then, the cells were washed from secondary antibodies with a solution of 0.1% Tween 20 3 times for 5 min. To stain the nuclei, the mixture was incubated with DAPI 100 ng/mL for 10 min in the dark at room temperature and then washed with PBS solution once. PBS was removed and glycerol was applied to the sample prior to covering with a coverslip. The stained proteins were visualized using a fluorescence microscope (Nikon Eclipse Ni-E, Japan). The number of γH2AX foci was calculated using ImageJ software with FindFoci plugins. 60–200 cells were analyzed in each sample.

### DNA isolation and bisulfite conversion

Cells (~ 10^6^) were resuspended in a lysis buffer (10 mM Tris pH = 8.0, NaCl 100 mM, 10 mM EDTA pH = 8.0, 0.5% SDS) and incubated with Proteinase K overnight. For DNA purification the phenol chloroform extraction was used. The DNA was then bisulfite converted using the EpiMark^®^ Bisulfite Conversion Kit (E3318S, NEB) according to the manufacturer's protocol.

### Infinium methylation EPIC Beadchip array

All DNA methylation experiments were performed according to Illumina manufacturer instructions for the Infinium Methylation EPIC 850K BeadChip Array (Illumina, USA). EPIC BeadChips were imaged using the Illumina iScan System (Illumina, United States).

### Isolation of RNA and cDNA synthesis

Isolation of RNA from cells was performed using the Lira reagent (Biolabmix, Russia) according to the manufacturer's protocol. The quality of the isolated RNA was assessed using agarose gel electrophoresis. The concentration of the resulting RNA preparation was measured spectrophotometrically on an Infinite 200 Pro M Plex plate reader (Tecan, Switzerland) determining the absorbance at 260 nm. The purity of the preparation was assessed by the ratio of absorption at wavelengths of 260 and 230 nm.

A mixture was prepared containing 1 μg of RNA, 1 U DNase I (Thermo Fisher Scientific, USA), and a reaction buffer with MgCl_2_. Incubation was carried out for 30 min at 37 ℃. Then, 1 μL of 50 μM EDTA was added and the solution was heated to 65 ℃ to inactivate DNase.

The first strand of cDNA was synthesized from a single-stranded RNA template using the MMLV RT kit (Evrogen, Russia) according to the manufacturer's protocol.

### RNA sequencing

Library preparation was performed with NEBNext Poly(A) mRNA Magnetic Isolation Module and NEBNext Ultra II Directional RNA Library Prep Kit (NEB) according to the manufacturer’s protocol. The library underwent a final cleanup using the Agencourt AMPure XP system (Beckman Coulter) after which the libraries’ size distribution and quality were assessed using a high-sensitivity DNA chip (Agilent Technologies). Libraries were subsequently quantified by Quant-iT DNA Assay Kit, High Sensitivity (ThermoFisher). Finally, libraries were sequenced by a high-throughput run on the Illumina HiSeq 2500 using 2 × 125 bp paired-end reads.

### Real-time PCR

Real-time PCR was performed in a volume of 20 μL using the obtained cDNA as a template, qPCRmix-HS SYBR (Evrogen, Russia) containing HS Taq DNA polymerase, SYBR Green I dye, a mixture of deoxynucleoside triphosphates, Mg^2+^, reaction buffer, and primers for the target gene (TMPRSS2_ERG_fwd-CAGGAGGCGGAGGCGGA and TMPRSS2_ERG_rev-GGCGTTGTAGCTGGGGGTGAG; dNp63_fwd-GAAAACAATGCCCAGACTCAA and dNp63_re-TGCGCGTGGTCTGTGTTA; TRIM29_fwd-AAAGGCTATCCCTCCCTCAT and TRIM29_rev-TAGAATGGCCGGTAGTGAGA) and a reference gene (ACTB_fwd—CACCATTGGCAATGAGCGGTTC and ACTB_rev—AGGTCTTTGCGGATGTCCACGT). Each sample was prepared four times.

### MSRE-PCR

The protocol was used from the article (Melnikov et al. 2005) with minor modifications. HpaII was used as a methyl-sensitive restriction enzyme. 500 ng genomic DNA was incubated with a restriction enzyme overnight. The treated DNA was purified by phenol–chloroform extraction. The purified DNA was used as a template for quantitative analysis. The region of the GAPDH gene in which there are no HpaII restriction sites was used as a control.

### Statistics or data analysis

Results were analyzed using two-tailed Student’s t-test, Fisher’s exact test and two-way ANOVA. P value less than 0.05 was considered statistically significant.

### Supplementary Information

**Additional file 1: Table S1.** related to Figure 1. Data sets used in the study. Sheet1—List of all datasets used in the study. Sheet2—List of PRAD sample IDs used in the study. Table S2 related to Figure 2. TP63 regulates the gene cluster associated with epigenetic variability and chromosomal instability in PRAD. Sheet1—list of genes from TP63 cluster. Sheet2—Ontology analysis of the TP63 cluster genes. Gene Ontology (GO) terms related to biological process. Sheet3—Ontology analysis of the TP63 cluster genes. Gene Ontology (GO) terms related to cellular components. Table S3 related to Figure 3. CpG sites associated with TP63 cluster belong to TP63-dependent enhancers and super-enhancer. Sheet1—list of CpG sites associated with TP63 cluster. Sheet2—Ontology analysis of genes located near the TP63 CpG sites. Gene Ontology (GO) terms related to biological process. Sheet3—Ontology analysis of genes located near the TP63 CpG sites. Disease ontology. Table S4 related to Figure 4. TRIM29 interacts with TP63 and regulates expression of the TP63 cluster. Sheet1—Ontology analysis of the TP63 cluster genes under TP63 and TRIM29 simultaneously regulation. Gene Ontology (GO) terms related to biological process. Table S5 related to Figure 5. TRIM29 promotes decrease of chromosomal instability in PRAD. Sheet1—Partners of TRIM29 in RWPE-1 cells. Sheet2—Association between the TP63 cluster and the response to genotoxic stress.** Figure S1.** related to Figure 2**. A** GSEA plots evaluating the TP63 cluster signatures upon differential expressing genes between cancer and normal samples in TCGA dataset. **B** Boxenplot represents expression level of *TP63* in TCGA PRAD samples. Each successive level outward 50%-percentile contains half of the remaining data. **C** Expression of *TP63* in PRAD and normal prostate epithelium cell lines. **D** Immunoblot (IB) analysis of overexpression (OE) of *TP63* in PC3 cells and knockdown (KD) of *TP63* in the RWPE-1 cells.** Figure S2.** related to Figure 3**. A** Representation of DNA methylation level of 1645 TP63 CpG sites in normal and PRAD cell lines. **B** Overexpression of *TP63* in PC3 promotes decreased methylation of two CpG sites (MRSE-qPCR) compared to an empty vector control. Error bar = SD. N = 3 **C** *TP63* knockdown in the RWPE-1 cell line promotes increased methylation of two CpG-sites (MRSE-qPCR) compared to control. Error Bar = SD. N = 3. **Figure S3.** related to Figure 4**.** **A** GSEA plots evaluating the TP63 cluster signature upon *TRIM29* knockdown in MCF10A (breast basal epithelium, GSE26238). **B** TP63 ChIP in RWPE-1 and PC3 cells with *TP63* overexpression demonstrates enrichment of *TRIM29* enhancer 1. All samples were done in triplicate. Error Bars = SEM IP - immunoprecipitation. **Figure S4.** Molecular mapping of TRIM29 and TP63 interactions. **A** Outline of the constructs of full length TRIM29 and of its truncated mutants. Immunoblotting (IB) of purified FLAG-tagged TRIM29 with anti-TP63 antibodies. **B**. Outline of the constructs of full length TP63 and of its truncated mutants. Immunoblotting (IB) of purified FLAG-tagged TP63 with anti-GFP antibodies. **Figure S5.** Violin plots of the γH2AX foci number per nucleus in RWPE-1 and RWPE-1 with knockdown of TRIM29 cells before and after TNFα (100 ng/mL) treatment. “***” stands for p-value<1e-8. *ns* non significant.

## Data Availability

ChIP-seq, RNA-seq and DNA methylation data reported in this paper have been deposited in GEO with accession number GSE204813.

## Abbreviations

TCGA: The Cancer Genome Atlas
ICGC: International Cancer Genome Consortium
WGCNA: Weighted gene co-expression network analysis
PRAD: Prostate adenocarcinoma

## References

[1] Sung H, Ferlay J, Siegel RL, Laversanne M, Soerjomataram I, Jemal A (2021). Global cancer statistics 2020: GLOBOCAN Estimates of incidence and mortality worldwide for 36 cancers in 185 Countries. CA Cancer J Clin.

[2] Kluth M, Hesse J, Heinl A, Krohn A, Steurer S, Sirma H (2013). Genomic deletion of MAP3K7 at 6q12-22 is associated with early PSA recurrence in prostate cancer and absence of TMPRSS2:ERG fusions. Mod Pathol.

[3] Taylor BS, Schultz N, Hieronymus H, Gopalan A, Xiao Y, Carver BS (2010). Integrative genomic profiling of human prostate cancer. Cancer Cell.

[4] Cancer Genome Atlas Research Network (2015). The molecular taxonomy of primary prostate cancer. Cell.

[5] Kgatle MM, Kalla AA, Islam MM, Sathekge M, Moorad R (2016). Prostate cancer: epigenetic alterations, risk factors, and therapy. Prostate Cancer.

[6] Ruggero K, Farran-Matas S, Martinez-Tebar A, Aytes A (2018). Epigenetic Regulation in prostate cancer progression. Curr Mol Biol Rep.

[7] Ferguson LR, Chen H, Collins AR, Connell M, Damia G, Dasgupta S (2015). Genomic instability in human cancer: molecular insights and opportunities for therapeutic attack and prevention through diet and nutrition. Semin Cancer Biol.

[8] Zhao SG, Chen WS, Li H, Foye A, Zhang M, Sjöström M (2020). The DNA methylation landscape of advanced prostate cancer. Nat Genet.

[9] Stelloo S, Nevedomskaya E, Kim Y, Schuurman K, Valle-Encinas E, Lobo J (2018). Integrative epigenetic taxonomy of primary prostate cancer. Nat Commun.

[10] Börno ST, Fischer A, Kerick M, Fälth M, Laible M, Brase JC (2012). Genome-wide DNA methylation events in TMPRSS2-ERG fusion-negative prostate cancers implicate an EZH2-dependent mechanism with miR-26a hypermethylation. Cancer Discov.

[11] Kobayashi Y, Absher DM, Gulzar ZG, Young SR, McKenney JK, Peehl DM (2011). DNA methylation profiling reveals novel biomarkers and important roles for DNA methyltransferases in prostate cancer. Genome Res.

[12] Stopsack KH, Whittaker CA, Gerke TA, Loda M, Kantoff PW, Mucci LA (2019). Aneuploidy drives lethal progression in prostate cancer. Proc Natl Acad Sci U S A.

[13] Babu D, Fullwood MJ. Expanding the effects of ERG on chromatin landscapes and dysregulated transcription in prostate cancer. Nat Genet. 2017;49:1294–5. 10.1038/ng.3944 28854182

[14] Kron KJ, Murison A, Zhou S, Huang V, Yamaguchi TN, Shiah Y-J, et al. TMPRSS2–ERG fusion co-opts master transcription factors and activates NOTCH signaling in primary prostate cancer. Nat Genet. 2017;49:1336–45. 10.1038/ng.3930 28783165

[15] Guo H, Wu Y, Nouri M, Spisak S, Russo JW, Sowalsky AG (2021). Androgen receptor and MYC equilibration centralizes on developmental super-enhancer. Nat Commun.

[16] Langfelder P, Horvath S (2008). WGCNA: an R package for weighted correlation network analysis. BMC Bioinformatics.

[17] Hou Y, Hu J, Zhou L, Liu L, Chen K, Yang X (2021). Integrative analysis of methylation and copy number variations of prostate adenocarcinoma based on weighted gene co-expression network analysis. Front Oncol.

[18] Ohandjo AQ, Liu Z, Dammer EB, Dill CD, Griffen TL, Carey KM (2019). Transcriptome network analysis identifies CXCL13-CXCR5 signaling modules in the prostate tumor immune microenvironment. Sci Rep.

[19] Li S, Li B, Zheng Y, Li M, Shi L, Pu X (2017). Exploring functions of long noncoding RNAs across multiple cancers through co-expression network. Sci Rep.

[20] Subramanian A, Tamayo P, Mootha VK, Mukherjee S, Ebert BL, Gillette MA (2005). Gene set enrichment analysis: a knowledge-based approach for interpreting genome-wide expression profiles. Proc Natl Acad Sci U S A.

[21] Pellacani D, Droop AP, Frame FM, Simms MS, Mann VM, Collins AT (2018). Phenotype-independent DNA methylation changes in prostate cancer. Br J Cancer.

[22] Henry GH, Malewska A, Joseph DB, Malladi VS, Lee J, Torrealba J (2018). A cellular anatomy of the normal adult human prostate and prostatic urethra. Cell Rep.

[23] Hnisz D, Day DS, Young RA (2016). Insulated neighborhoods: structural and functional units of mammalian gene control. Cell.

[24] Keenan AB, Torre D, Lachmann A, Leong AK, Wojciechowicz ML, Utti V (2019). ChEA3: transcription factor enrichment analysis by orthogonal omics integration. Nucleic Acids Res.

[25] Somerville TDD, Xu Y, Miyabayashi K, Tiriac H, Cleary CR, Maia-Silva D (2018). TP63-Mediated enhancer reprogramming drives the squamous subtype of pancreatic ductal adenocarcinoma. Cell Rep.

[26] Jiang Y-Y, Jiang Y, Li C-Q, Zhang Y, Dakle P, Kaur H (2020). TP63, SOX2, and KLF5 establish a core regulatory circuitry that controls epigenetic and transcription patterns in esophageal squamous cell carcinoma cell lines. Gastroenterology.

[27] Jiang Y, Jiang Y-Y, Xie J-J, Mayakonda A, Hazawa M, Chen L (2018). Co-activation of super-enhancer-driven CCAT1 by TP63 and SOX2 promotes squamous cancer progression. Nat Commun.

[28] Kouwenhoven EN, Oti M, Niehues H, van Heeringen SJ, Schalkwijk J, Stunnenberg HG (2015). Transcription factor p63 bookmarks and regulates dynamic enhancers during epidermal differentiation. EMBO Rep.

[29] Wu T, Hu E, Xu S, Chen M, Guo P, Dai Z (2021). clusterProfiler 4.0: A universal enrichment tool for interpreting omics data. Innovation.

[30] Fulco CP, Nasser J, Jones TR, Munson G, Bergman DT, Subramanian V (2019). Activity-by-contact model of enhancer-promoter regulation from thousands of CRISPR perturbations. Nat Genet.

[31] Yi M, Tan Y, Wang L, Cai J, Li X, Zeng Z (2020). TP63 links chromatin remodeling and enhancer reprogramming to epidermal differentiation and squamous cell carcinoma development. Cell Mol Life Sci.

[32] Zhou Y, Liu H, Wang J, Wang X, Qian L, Xu F (2020). ΔNp63α exerts antitumor functions in cervical squamous cell carcinoma. Oncogene.

[33] Yuan Z, Villagra A, Peng L, Coppola D, Glozak M, Sotomayor EM (2010). The ATDC (TRIM29) protein binds p53 and antagonizes p53-mediated functions. Mol Cell Biol.

[34] Fraser M, Sabelnykova VY, Yamaguchi TN, Heisler LE, Livingstone J, Huang V (2017). Genomic hallmarks of localized, non-indolent prostate cancer. Nature.

[35] Zhang J, Bajari R, Andric D, Gerthoffert F, Lepsa A, Nahal-Bose H (2019). The international cancer genome consortium data portal. Nat Biotechnol.

[36] Palmbos PL, Wang Y, Bankhead III A, Kelleher AJ, Wang L, Yang H (2019). ATDC mediates a TP63-regulated basal cancer invasive program. Oncogene.

[37] Li Q, Lin L, Tong Y, Liu Y, Mou J, Wang X (2018). TRIM29 negatively controls antiviral immune response through targeting STING for degradation. Cell Discov.

[38] Cao Y, Shi L, Wang M, Hou J, Wei Y, Du C (2019). ATDC contributes to sustaining the growth and invasion of glioma cells through regulating Wnt/β-catenin signaling. Chem Biol Interact.

[39] Xing J, Weng L, Yuan B, Wang Z, Jia L, Jin R (2016). Identification of a role for TRIM29 in the control of innate immunity in the respiratory tract. Nat Immunol.

[40] Olivieri M, Cho T, Álvarez-Quilón A, Li K, Schellenberg MJ, Zimmermann M (2020). A genetic map of the response to DNA damage in human cells. Cell.

[41] Schleicher EM, Dhoonmoon A, Jackson LM, Clements KE, Stump CL, Nicolae CM (2020). Dual genome-wide CRISPR knockout and CRISPR activation screens identify mechanisms that regulate the resistance to multiple ATR inhibitors. PLoS Genet.

[42] Masuda Y, Takahashi H, Sato S, Tomomori-Sato C, Saraf A, Washburn MP (2015). TRIM29 regulates the assembly of DNA repair proteins into damaged chromatin. Nat Commun.

[43] Wang L, Yang H, Palmbos PL, Ney G, Detzler TA, Coleman D (2014). ATDC/TRIM29 phosphorylation by ATM/MAPKAP kinase 2 mediates radioresistance in pancreatic cancer cells. Cancer Res.

[44] Tomlins SA, Rhodes DR, Perner S, Dhanasekaran SM, Mehra R, Sun X-W (2005). Recurrent fusion of TMPRSS2 and ETS transcription factor genes in prostate cancer. Science.

[45] Mani RS, Amin MA, Li X, Kalyana-Sundaram S, Veeneman BA, Wang L (2016). Inflammation-induced oxidative stress mediates gene fusion formation in prostate cancer. Cell Rep.

[46] Soares E, Zhou H (2018). Master regulatory role of p63 in epidermal development and disease. Cell Mol Life Sci.

[47] Hatakeyama S (2016). Early evidence for the role of TRIM29 in multiple cancer models. Expert Opin Ther Targets.

[48] Kanno Y, Watanabe M, Kimura T, Nonomura K, Tanaka S, Hatakeyama S (2014). TRIM29 as a novel prostate basal cell marker for diagnosis of prostate cancer. Acta Histochem.

[49] Chu Y, Yang X (2011). SUMO E3 ligase activity of TRIM proteins. Oncogene.

[50] Peschiaroli A, Scialpi F, Bernassola F, El Sherbini ES, Melino G (2010). The E3 ubiquitin ligase WWP1 regulates ΔNp63-dependent transcription through Lys63 linkages. Biochem Biophys Res Commun.

[51] Landré V, Revi B, Mir MG, Verma C, Hupp TR, Gilbert N (2017). Regulation of transcriptional activators by DNA-binding domain ubiquitination. Cell Death Differ.

[52] Ranieri M, Vivo M, De Simone M, Guerrini L, Pollice A, La Mantia G (2018). Sumoylation and ubiquitylation crosstalk in the control of ΔNp63α protein stability. Gene.

[53] Bao X, Rubin AJ, Qu K, Zhang J, Giresi PG, Chang HY (2015). A novel ATAC-seq approach reveals lineage-specific reinforcement of the open chromatin landscape via cooperation between BAF and p63. Genome Biol.

[54] Rinaldi L, Datta D, Serrat J, Morey L, Solanas G, Avgustinova A (2016). Dnmt3a and Dnmt3b associate with enhancers to regulate human epidermal stem cell homeostasis. Cell Stem Cell.

[55] Babalyan K, Sultanov R, Generozov E, Sharova E, Kostryukova E, Larin A (2018). LogLoss-BERAF: An ensemble-based machine learning model for constructing highly accurate diagnostic sets of methylation sites accounting for heterogeneity in prostate cancer. PLoS ONE.

[56] Assenov Y, Müller F, Lutsik P, Walter J, Lengauer T, Bock C (2014). Comprehensive analysis of DNA methylation data with RnBeads. Nat Methods.

[57] Davis S, Du P, Bilke S, Triche, Jr. T, Bootwalla M (2023). methylumi: Handle Illumina methylation data. 10.18129/B9.bioc.methylumi, R package version 2.48.0, https://bioconductor.org/packages/methylumi..

[58] Teschendorff AE, Marabita F, Lechner M, Bartlett T, Tegner J, Gomez-Cabrero D (2013). A beta-mixture quantile normalization method for correcting probe design bias in Illumina Infinium 450 k DNA methylation data. Bioinformatics.

[59] Xu Z, Niu L, Li L, Taylor JA (2016). ENmix: a novel background correction method for Illumina HumanMethylation450 BeadChip. Nucleic Acids Res.

[60] Ritchie ME, Phipson B, Wu D, Hu Y, Law CW, Shi W (2015). limma powers differential expression analyses for RNA-sequencing and microarray studies. Nucleic Acids Res.

[61] Heinz S, Benner C, Spann N, Bertolino E, Lin YC, Laslo P (2010). Simple combinations of lineage-determining transcription factors prime cis-regulatory elements required for macrophage and B cell identities. Mol Cell.

[62] Kulakovskiy IV, Vorontsov IE, Yevshin IS, Sharipov RN, Fedorova AD, Rumynskiy EI (2018). HOCOMOCO: towards a complete collection of transcription factor binding models for human and mouse via large-scale ChIP-Seq analysis. Nucleic Acids Res.

[63] Wang X, Park J, Susztak K, Zhang NR, Li M (2019). Bulk tissue cell type deconvolution with multi-subject single-cell expression reference. Nat Commun.

[64] Bolger AM, Lohse M, Usadel B (2014). Trimmomatic: a flexible trimmer for Illumina sequence data. Bioinformatics.

[65] Li H, Durbin R (2009). Fast and accurate short read alignment with burrows-wheeler transform. Bioinformatics.

[66] Zhang Y, Liu T, Meyer CA, Eeckhoute J, Johnson DS, Bernstein BE (2008). Model-based analysis of ChIP-Seq (MACS). Genome Biol.

[67] Ramírez F, Ryan DP, Grüning B, Bhardwaj V, Kilpert F, Richter AS (2016). deepTools2: a next generation web server for deep-sequencing data analysis. Nucleic Acids Res.

[68] Stop and Go Extraction Tips for Matrix-assisted Laser Desorption/Ionization Nanoelectrospray and LC/MS Sample Pretreatment in Proteomics Analytical Chemistry 2003;75(3):663-670 10.1021/ac026117i 10.1021/ac026117i 12585499

