# Supplementary figures and images for: TP63–TRIM29 axis regulates enhancer methylation and chromosomal instability in prostate cancer
Source: Epigenetics Chromatin. 2024 Mar 14;17:6. doi: 10.1186/s13072-024-00529-7 (PMC10938740; doi:10.1186/s13072-024-00529-7)

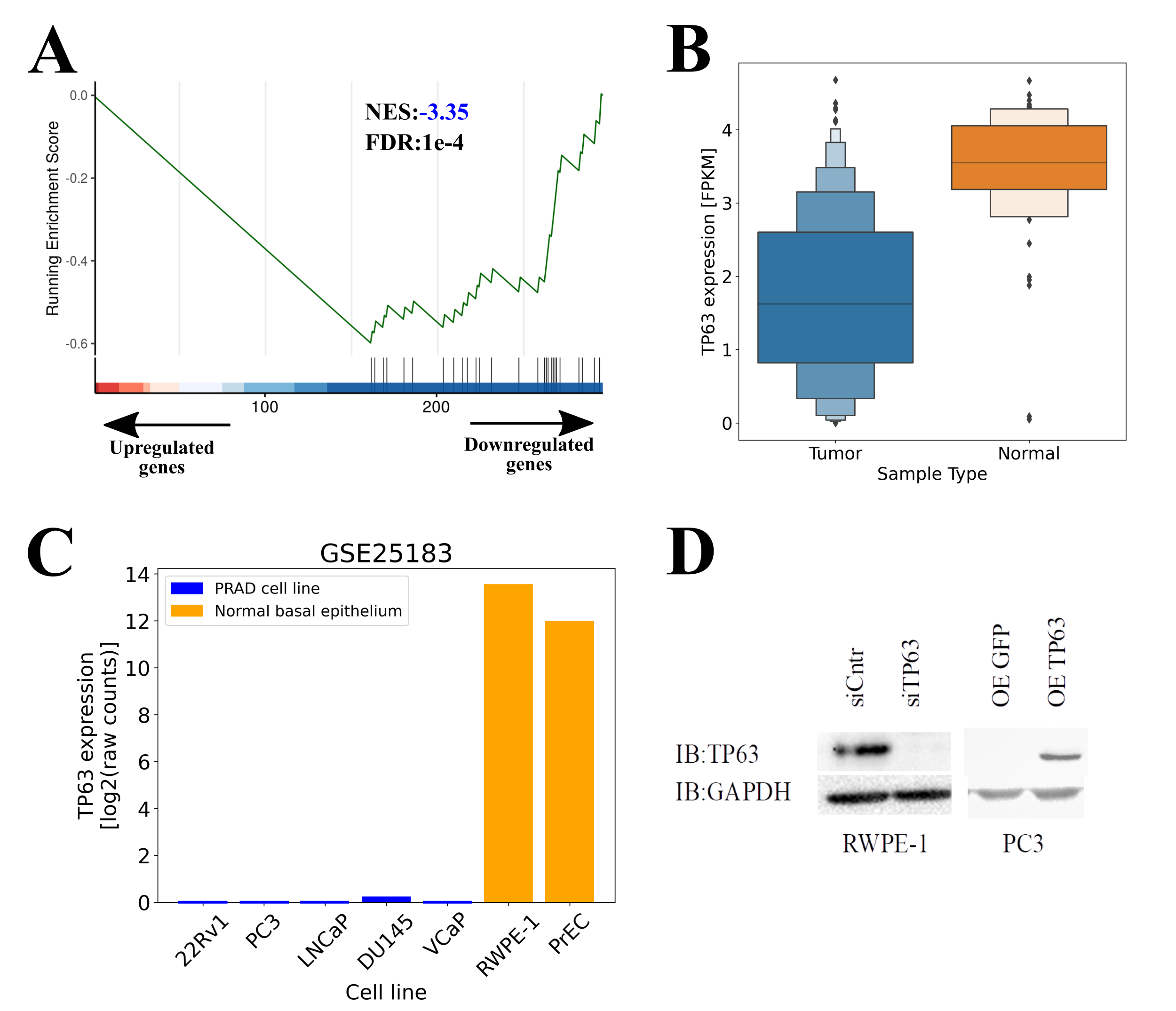

Supplement: Supplementary file 1 — Additional file 1: Table S1. related to Figure 1. Data sets used in the study. Sheet1—List of all datasets used in the study. Sheet2—List of PRAD sample IDs used in the study. Table S2 related to Figure 2. TP63 regulates the gene cluster associated with epigenetic variability and chromosomal instability in PRAD. Sheet1—list of genes from TP63 cluster. Sheet2—Ontology analysis of the TP63 cluster genes. Gene Ontology (GO) terms related to biological process. Sheet3—Ontology analysis of the TP63 cluster genes. Gene Ontology (GO) terms related to cellular components. Table S3 related to Figure 3. CpG sites associated with TP63 cluster belong to TP63-dependent enhancers and super-enhancer. Sheet1—list of CpG sites associated with TP63 cluster. Sheet2—Ontology analysis of genes located near the TP63 CpG sites. Gene Ontology (GO) terms related to biological process. Sheet3—Ontology analysis of genes located near the TP63 CpG sites. Disease ontology. Table S4 related to Figure 4. TRIM29 interacts with TP63 and regulates expression of the TP63 cluster. Sheet1—Ontology analysis of the TP63 cluster genes under TP63 and TRIM29 simultaneously regulation. Gene Ontology (GO) terms related to biological process. Table S5 related to Figure 5. TRIM29 promotes decrease of chromosomal instability in PRAD. Sheet1—Partners of TRIM29 in RWPE-1 cells. Sheet2—Association between the TP63 cluster and the response to genotoxic stress. Figure S1. related to Figure 2. A GSEA plots evaluating the TP63 cluster signatures upon differential expressing genes between cancer and normal samples in TCGA dataset. B Boxenplot represents expression level of TP63 in TCGA PRAD samples. Each successive level outward 50%-percentile contains half of the remaining data. C Expression of TP63 in PRAD and normal prostate epithelium cell lines. D Immunoblot (IB) analysis of overexpression (OE) of TP63 in PC3 cells and knockdown (KD) of TP63 in the RWPE-1 cells. Figure S2. related to Figure 3. A Representation of DN [file 13072_2024_529_MOESM1_ESM.zip › New folder/FigureS1.jpg]

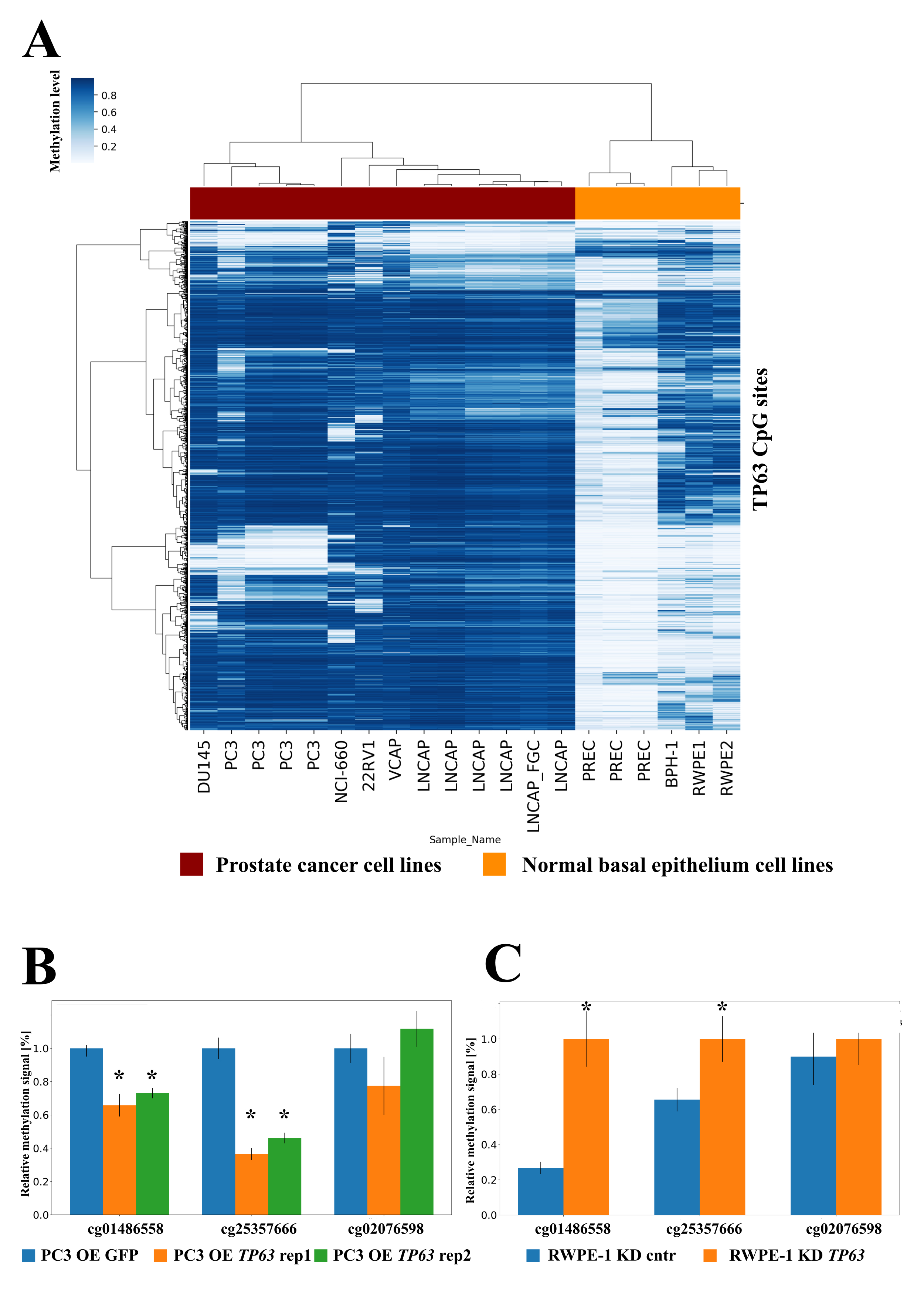

Supplement: Supplementary file 1 — Additional file 1: Table S1. related to Figure 1. Data sets used in the study. Sheet1—List of all datasets used in the study. Sheet2—List of PRAD sample IDs used in the study. Table S2 related to Figure 2. TP63 regulates the gene cluster associated with epigenetic variability and chromosomal instability in PRAD. Sheet1—list of genes from TP63 cluster. Sheet2—Ontology analysis of the TP63 cluster genes. Gene Ontology (GO) terms related to biological process. Sheet3—Ontology analysis of the TP63 cluster genes. Gene Ontology (GO) terms related to cellular components. Table S3 related to Figure 3. CpG sites associated with TP63 cluster belong to TP63-dependent enhancers and super-enhancer. Sheet1—list of CpG sites associated with TP63 cluster. Sheet2—Ontology analysis of genes located near the TP63 CpG sites. Gene Ontology (GO) terms related to biological process. Sheet3—Ontology analysis of genes located near the TP63 CpG sites. Disease ontology. Table S4 related to Figure 4. TRIM29 interacts with TP63 and regulates expression of the TP63 cluster. Sheet1—Ontology analysis of the TP63 cluster genes under TP63 and TRIM29 simultaneously regulation. Gene Ontology (GO) terms related to biological process. Table S5 related to Figure 5. TRIM29 promotes decrease of chromosomal instability in PRAD. Sheet1—Partners of TRIM29 in RWPE-1 cells. Sheet2—Association between the TP63 cluster and the response to genotoxic stress. Figure S1. related to Figure 2. A GSEA plots evaluating the TP63 cluster signatures upon differential expressing genes between cancer and normal samples in TCGA dataset. B Boxenplot represents expression level of TP63 in TCGA PRAD samples. Each successive level outward 50%-percentile contains half of the remaining data. C Expression of TP63 in PRAD and normal prostate epithelium cell lines. D Immunoblot (IB) analysis of overexpression (OE) of TP63 in PC3 cells and knockdown (KD) of TP63 in the RWPE-1 cells. Figure S2. related to Figure 3. A Representation of DN [file 13072_2024_529_MOESM1_ESM.zip › New folder/FigureS2.jpg]

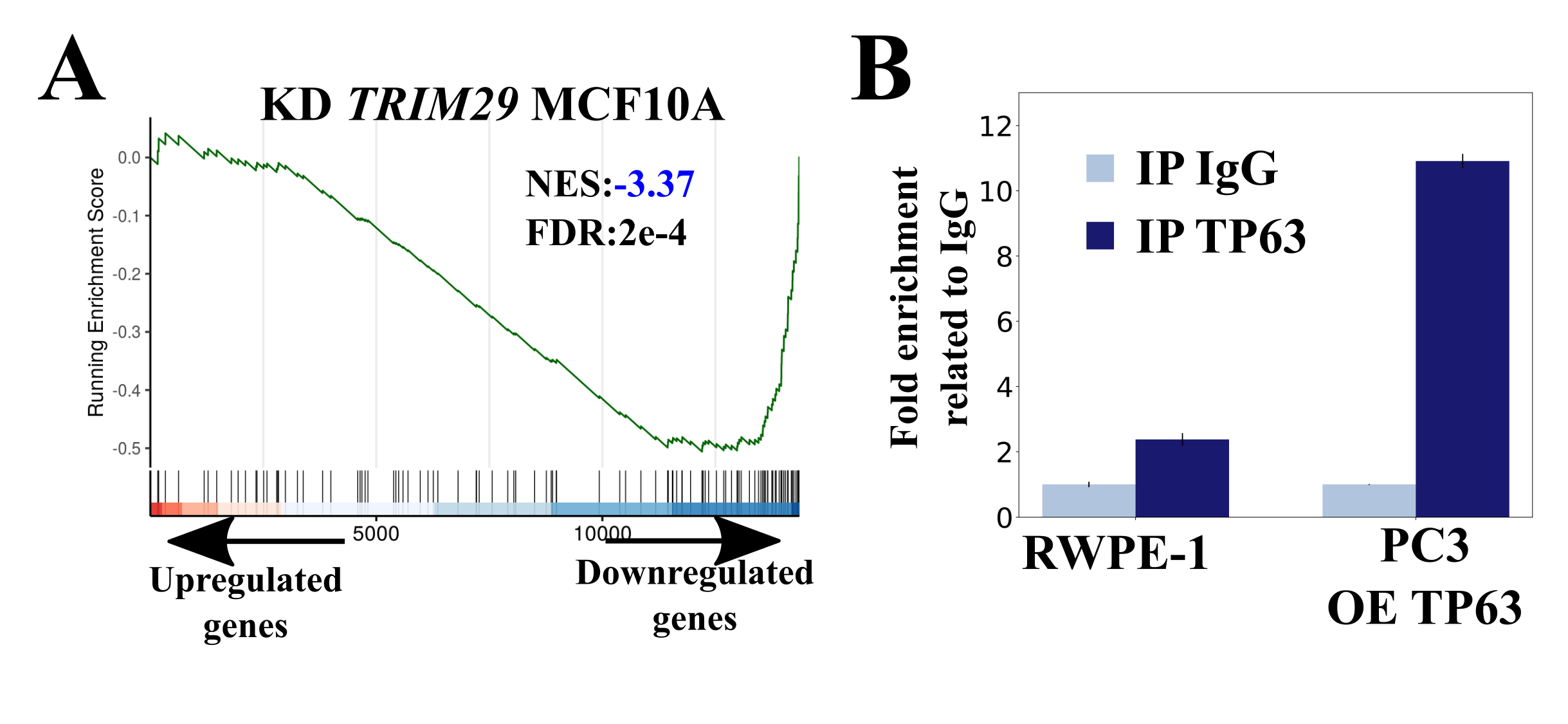

Supplement: Supplementary file 1 — Additional file 1: Table S1. related to Figure 1. Data sets used in the study. Sheet1—List of all datasets used in the study. Sheet2—List of PRAD sample IDs used in the study. Table S2 related to Figure 2. TP63 regulates the gene cluster associated with epigenetic variability and chromosomal instability in PRAD. Sheet1—list of genes from TP63 cluster. Sheet2—Ontology analysis of the TP63 cluster genes. Gene Ontology (GO) terms related to biological process. Sheet3—Ontology analysis of the TP63 cluster genes. Gene Ontology (GO) terms related to cellular components. Table S3 related to Figure 3. CpG sites associated with TP63 cluster belong to TP63-dependent enhancers and super-enhancer. Sheet1—list of CpG sites associated with TP63 cluster. Sheet2—Ontology analysis of genes located near the TP63 CpG sites. Gene Ontology (GO) terms related to biological process. Sheet3—Ontology analysis of genes located near the TP63 CpG sites. Disease ontology. Table S4 related to Figure 4. TRIM29 interacts with TP63 and regulates expression of the TP63 cluster. Sheet1—Ontology analysis of the TP63 cluster genes under TP63 and TRIM29 simultaneously regulation. Gene Ontology (GO) terms related to biological process. Table S5 related to Figure 5. TRIM29 promotes decrease of chromosomal instability in PRAD. Sheet1—Partners of TRIM29 in RWPE-1 cells. Sheet2—Association between the TP63 cluster and the response to genotoxic stress. Figure S1. related to Figure 2. A GSEA plots evaluating the TP63 cluster signatures upon differential expressing genes between cancer and normal samples in TCGA dataset. B Boxenplot represents expression level of TP63 in TCGA PRAD samples. Each successive level outward 50%-percentile contains half of the remaining data. C Expression of TP63 in PRAD and normal prostate epithelium cell lines. D Immunoblot (IB) analysis of overexpression (OE) of TP63 in PC3 cells and knockdown (KD) of TP63 in the RWPE-1 cells. Figure S2. related to Figure 3. A Representation of DN [file 13072_2024_529_MOESM1_ESM.zip › New folder/FigureS3.jpg]

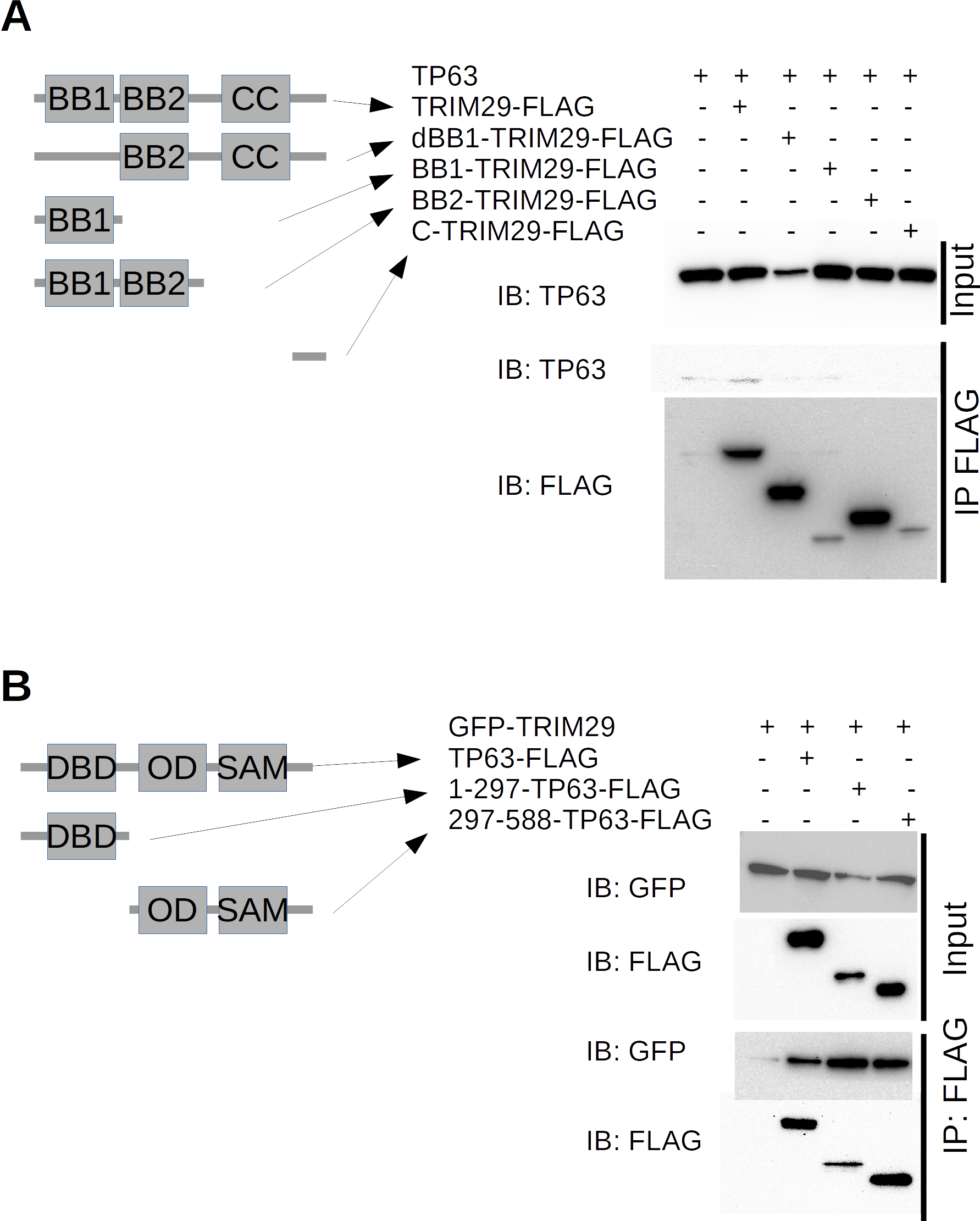

Supplement: Supplementary file 1 — Additional file 1: Table S1. related to Figure 1. Data sets used in the study. Sheet1—List of all datasets used in the study. Sheet2—List of PRAD sample IDs used in the study. Table S2 related to Figure 2. TP63 regulates the gene cluster associated with epigenetic variability and chromosomal instability in PRAD. Sheet1—list of genes from TP63 cluster. Sheet2—Ontology analysis of the TP63 cluster genes. Gene Ontology (GO) terms related to biological process. Sheet3—Ontology analysis of the TP63 cluster genes. Gene Ontology (GO) terms related to cellular components. Table S3 related to Figure 3. CpG sites associated with TP63 cluster belong to TP63-dependent enhancers and super-enhancer. Sheet1—list of CpG sites associated with TP63 cluster. Sheet2—Ontology analysis of genes located near the TP63 CpG sites. Gene Ontology (GO) terms related to biological process. Sheet3—Ontology analysis of genes located near the TP63 CpG sites. Disease ontology. Table S4 related to Figure 4. TRIM29 interacts with TP63 and regulates expression of the TP63 cluster. Sheet1—Ontology analysis of the TP63 cluster genes under TP63 and TRIM29 simultaneously regulation. Gene Ontology (GO) terms related to biological process. Table S5 related to Figure 5. TRIM29 promotes decrease of chromosomal instability in PRAD. Sheet1—Partners of TRIM29 in RWPE-1 cells. Sheet2—Association between the TP63 cluster and the response to genotoxic stress. Figure S1. related to Figure 2. A GSEA plots evaluating the TP63 cluster signatures upon differential expressing genes between cancer and normal samples in TCGA dataset. B Boxenplot represents expression level of TP63 in TCGA PRAD samples. Each successive level outward 50%-percentile contains half of the remaining data. C Expression of TP63 in PRAD and normal prostate epithelium cell lines. D Immunoblot (IB) analysis of overexpression (OE) of TP63 in PC3 cells and knockdown (KD) of TP63 in the RWPE-1 cells. Figure S2. related to Figure 3. A Representation of DN [file 13072_2024_529_MOESM1_ESM.zip › New folder/FigureS4.jpg]

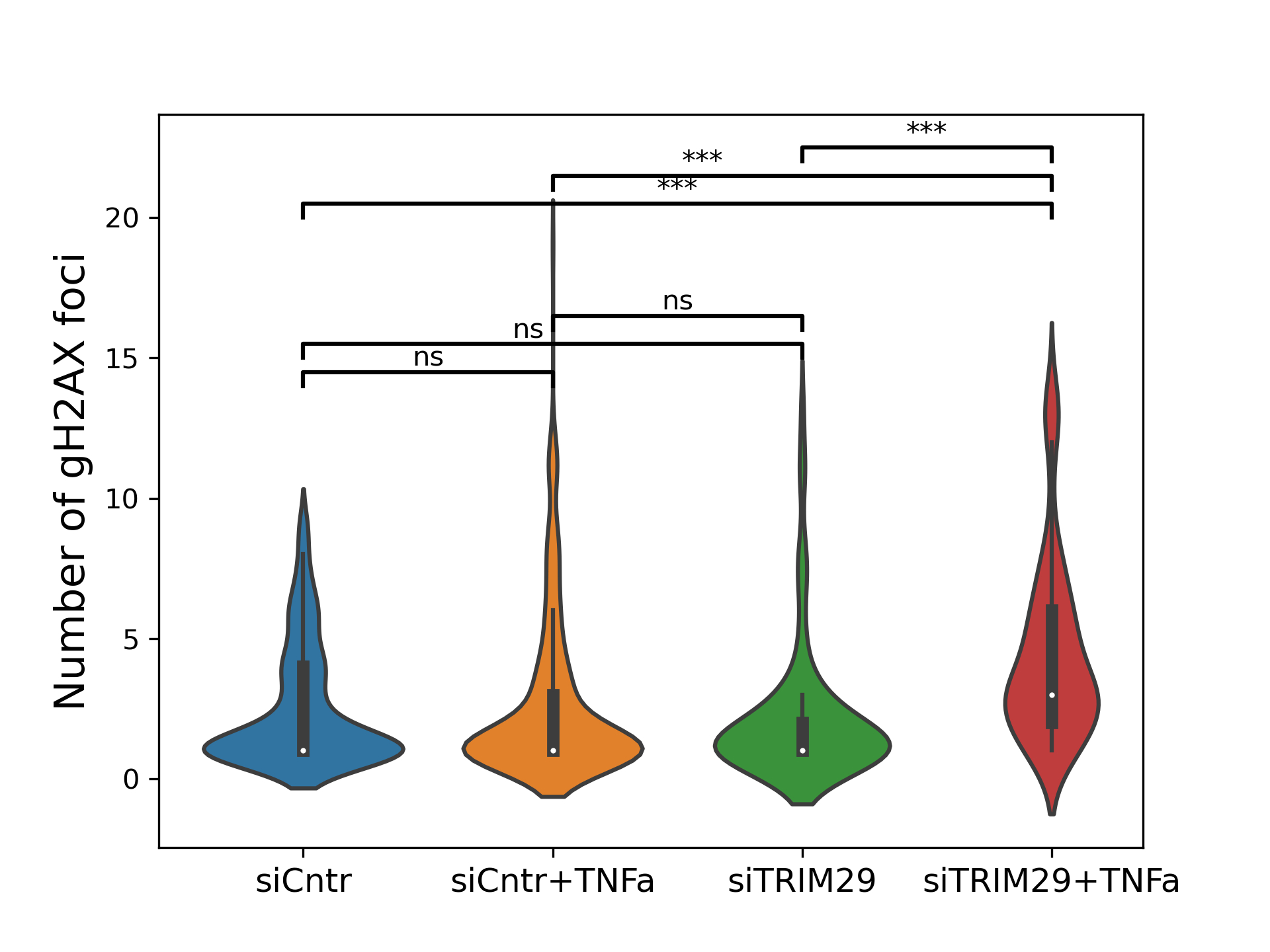

Supplement: Supplementary file 1 — Additional file 1: Table S1. related to Figure 1. Data sets used in the study. Sheet1—List of all datasets used in the study. Sheet2—List of PRAD sample IDs used in the study. Table S2 related to Figure 2. TP63 regulates the gene cluster associated with epigenetic variability and chromosomal instability in PRAD. Sheet1—list of genes from TP63 cluster. Sheet2—Ontology analysis of the TP63 cluster genes. Gene Ontology (GO) terms related to biological process. Sheet3—Ontology analysis of the TP63 cluster genes. Gene Ontology (GO) terms related to cellular components. Table S3 related to Figure 3. CpG sites associated with TP63 cluster belong to TP63-dependent enhancers and super-enhancer. Sheet1—list of CpG sites associated with TP63 cluster. Sheet2—Ontology analysis of genes located near the TP63 CpG sites. Gene Ontology (GO) terms related to biological process. Sheet3—Ontology analysis of genes located near the TP63 CpG sites. Disease ontology. Table S4 related to Figure 4. TRIM29 interacts with TP63 and regulates expression of the TP63 cluster. Sheet1—Ontology analysis of the TP63 cluster genes under TP63 and TRIM29 simultaneously regulation. Gene Ontology (GO) terms related to biological process. Table S5 related to Figure 5. TRIM29 promotes decrease of chromosomal instability in PRAD. Sheet1—Partners of TRIM29 in RWPE-1 cells. Sheet2—Association between the TP63 cluster and the response to genotoxic stress. Figure S1. related to Figure 2. A GSEA plots evaluating the TP63 cluster signatures upon differential expressing genes between cancer and normal samples in TCGA dataset. B Boxenplot represents expression level of TP63 in TCGA PRAD samples. Each successive level outward 50%-percentile contains half of the remaining data. C Expression of TP63 in PRAD and normal prostate epithelium cell lines. D Immunoblot (IB) analysis of overexpression (OE) of TP63 in PC3 cells and knockdown (KD) of TP63 in the RWPE-1 cells. Figure S2. related to Figure 3. A Representation of DN [file 13072_2024_529_MOESM1_ESM.zip › New folder/FigureS5.jpg]
